# PAD4 Immunization Triggers Anti-Citrullinated Peptide Antibodies in Normal Mice: Analysis With Peptide Arrays

**DOI:** 10.3389/fimmu.2022.840035

**Published:** 2022-03-31

**Authors:** Marie F. Hemon, Nathalie C. Lambert, Fanny Arnoux, Jean Roudier, Isabelle Auger

**Affiliations:** ^1^ Institut National de la Santé et de la Recherche Médicale (INSERM) Unité Mixte de Recherche (UMRs) 1097, Aix Marseille University, Marseille, France; ^2^ Arthritis R&D, Neuilly-sur-Seine, France; ^3^ Assistance Publique Hôpitaux de Marseille (APHM), Rhumatologie, Marseille, France

**Keywords:** rheumatoid arthritis, antibodies to citrullinated proteins, peptidyl arginine deiminase, peptide array, ACPA detection

## Abstract

The critical immunological event in rheumatoid arthritis (RA) is the production of antibodies to citrullinated proteins (ACPAs), ie proteins on which arginines have been transformed into citrullines by peptidyl arginine deiminases (PAD). In C3H mice, immunization with PAD4 triggers the production of ACPAs. Here, we developed a peptide array to analyze the fine specificity of anti-citrullinated peptide antibodies and used it to characterize the ACPA response after hPAD4 immunization in mice expressing different H-2 haplotypes. Sera from C3H, DBA/2, BALB/c and C57BL/6 mice immunized with human PAD4 (hPAD4) or control-matched mice immunized with phosphate buffered saline (PBS) were used to screen peptide arrays containing 169 peptides from collagen, filaggrin, EBNA, proteoglycan, enolase, alpha and beta fibrinogen, histon and vimentin. Human PAD4 immunization induced antibodies directed against numerous citrullinated peptides from fibrinogen, histon 4 and vimentin. Most peptides were recognized under their arginine and citrullinated forms. DBA/2 and BALB/c mice (H-2d) had the lowest anti-citrullinated peptide IgG responses. C3H (H-2k) and BL6 mice (H-2b) had the highest anti-citrullinated peptide IgG responses. The newly developed peptide array allows us to characterize the ACPA production after hPAD4 immunization in mice on the H-2d, H-2k or H-2b backgrounds. This sensitive tool will be useful for further studies on mice for prevention of ACPA production by PAD tolerization.

## Introduction

The sera of two thirds of patients with rheumatoid arthritis (RA) contain anti-citrullinated protein (ACPA) IgG autoantibodies (1). ACPAs recognize citrulline on numerous proteins like filaggrin, fibrin, vimentin, enolase, collagen… (2–5). Citrulline results from post translational modification of arginine, by peptidyl arginine deiminases (PADs). The T cells that help the development of an IgG immune response to multiple citrullinated proteins are poorly characterized and their target antigen(s) are unknown.

PAD4 may be a critical T cell target in the development of anti-citrullinated protein IgG autoantibodies. Indeed, i) autoantibodies to PAD4 are present during the preclinical phase of RA and are associated with ACPA (6), ii) PAD4 directly binds the many proteins it citrullinates. Thus, it can act as a carrier and potentially contribute to the development of IgG responses to the many proteins it binds, under native or citrullinated form (7).

We previously demonstrated this mechanism in a mouse model where C3H mice immunized with PADs developed antibodies and T cells to PAD and IgG antibodies to citrullinated fibrinogen peptides, in the absence of any T cell response to native or citrullinated fibrinogen (8).

Moreover, we observed the importance of the Major Histocompatibility Complex (MHC) background of the immunized mouse on the development of ACPAs. Indeed, C3H mice, which developed anti-citrullinated fibrinogen peptides after immunization with PADs, express an I-E beta k chain homologous to RA predisposing HLA-DRB1*04:01. Conversely, DBA/2 mice, which failed to develop antibodies to citrullinated fibrinogen peptides, express an I-E beta d chain homologous to non RA predisposing HLA-DRB1*04:02 (8).

Here, we undertook to analyze the fine specificity of the anti-citrullinated peptide response in C3H (H-2k), DBA/2 (H-2d), BALB/c (H-2d), BL6 (H-2b) mice immunized with hPAD4. Therefore, we developed a peptide array made of 33 arginine-containing peptides and their 136 citrulline-substituted variants from collagen, filaggrin, ebna 2, proteoglycan, enolase, alpha and beta fibrinogen, histon 4 and vimentin and used it to analyze sera from 20 mice immunized with human PAD4 and 21 control mice immunized with PBS.

## Materials and Methods

### Mice

We tested healthy wild type mice:

- C3H/HeNHsd (C3H) mice express an I-E beta k chain homologous to the RA-associated HLA-DRB1*04:01 allele (Envigo Laboratories, Gannat, France).
- DBA/2JRccHsd (DBA/2) and BALB/cOlaHsd (BALB/c) mice express an I-E beta d chain homologous to the “non-RA-associated” HLA-DRB1*04:02 allele (Envigo Laboratories, Gannat, France).
- C57BL/6NRj (BL6) mice express I-Ab and no I-E molecules (Janvier Labs, Le Genest-Saint-Isle, France).

All mice were 7-9 week old females, weighing 20 to 30 g, randomly divided into two groups (immunized by hPAD4 or PBS). Mice were housed at the Luminy INSERM Institute, Marseille (A1301303).

### Protein

Human PAD4 (hPAD4) was purchased from Proteogenix, Schiltigheim, France. Its activity and non citrullinated status were checked before immunization.

Purity of recombinant hPAD4 protein produced by Proteogenix was higher than 90%. Purity evaluation was made on SDS-PAGE gel using the GelAnalyzer software by Proteogenix. This software identifies each protein band on a track, calculates its intensity and subtracts the background to evaluate the protein of interest’s purity. To test the capacity of hPAD4 to citrullinate, native human fibrinogen was incubated in 0.1 M Tris HCl (pH7.4), 10 mM CaCl2, 5 mM dithiothreitol buffer with human PAD4 for 2 hours at 37°C. Citrullinated human fibrinogen was detected by ELISA using sera from ACPA positive RA patients.

The absence autocitrullination of hPAD4 was checked by i/using sera from ACPA positive RA patients in ELISA assays, ii/using the anti-modified citrulline Western blot detection kit, Millipore, France. We found that hPAD4 was not citrullinated.

### Immunization Protocol

Mice were immunized subcutaneously with 100 μg of human PAD4 or phosphate buffered saline (PBS) in Freund’s complete adjuvant (CFA). Three booster injections with 100 μg of human or PBS in Freund’ incomplete adjuvant (IFA) were given subcutaneously 15, 30 and 45 days later.

### Detection of Anti-PAD Antibodies by ELISA

Plates were coated with 0.5 μg of human PAD4 and blocked with bovine serum albumin. Sera from mice obtained at 15, 30, 45 and 60 days post first immunization were incubated. After washing with PBS, anti-mouse IgG-peroxidase was added. After tetramethyl benzidine incubation, optical density (OD) was read at 405 nm. Background OD was obtained by adding each serum to a well without protein. A positive serum was defined by a ratio test OD/background OD higher than 2.

### PepStar™ Peptides

PepStar™ peptide microarrays (JPT Peptide Technologies, Berlin, Germany) comprise 169 15-mers from the alpha and beta chain of fibrinogen, vimentin, histon 4, enolase, proteoglycan, filaggrin, ebna 2 and collagen (**Supplementary Figures 1**, **2**). Thirty three arginine-containing peptides and their 136 citrulline-substituted variants (**Supplementary Table 1**) were synthesized on cellulose membranes using SPOT™ synthesis technology (9). A reactivity tag was attached to the N-terminus of each peptide. The peptides were cleaved and eluted from the membrane. Quality control measurements using liquid chromatography mass spectrometry were performed. Peptides were immobilized on the epoxy-modified slide surfaces using chemoselective coupling. All peptides were deposited (10^−15^ moles) in three identical subarrays per block/slide enabling analysis of assay homogeneity and reliability of the results. Peptide microarrays were scanned after the printing process for identification and quality control of each individual spot.

### Detection of ACPAs by PepStar™ Peptide Arrays

The serum profiling experiment was performed on 41 mouse serum samples diluted 1:200 in blocking buffer (3% BSA, 0.1% Tween 20 in TBS-Buffer). Sera from 12 hPAD4 immunized mice and 9 PBS immunized mice were tested at day 45 post first immunization. Sera from 8 hPAD4 immunized mice and 12 PBS immunized mice were tested at day 60 post first immunization. Sera were incubated for 1 hour at 30°C on a multiwell microarray slide containing 20 individual mini-arrays. After a washing step in TBS-buffer including 0.1% Tween20, pH 7.2, fluorescently labeled secondary antibody (goat anti-mouse IgG, DyLight 650, Thermo Fisher Scientific, Illkirch-Graffenstaden, France) was added at 0.1 µg/ml for 1 hour. After washing in 50 mM TBS-buffer including 0.1% Tween20, pH 7.2 and drying, the array was scanned at 635 nm (Axon Genepix Scanner 4300 SL50, JPT Peptide Technologies, Berlin, Germany). Images were quantified using spot-recognition software GenePix (Molecular Devices). For each spot, mean signal intensity was extracted (between 0 and 65535 arbitrary units). For each mouse strain, mean background signal intensity was obtained for every peptide using all the sera from mice immunized with PBS. A positive serum was defined by a ratio (mean test signal/mean background signal) higher than 3. Ratios are listed in **Supplementary Figures 1**, **2**.

### Statistical Analysis

Comparisons between groups were performed using Fisher exact and Mann Whitney tests (GraphPad Prism 9.1.2 Software).

### Study Approval

All animal care and experimental procedures were performed in agreement with the Animal Ethics Committee of Marseille and the Ministère de l’Enseignement Supérieur et de la Recherche, France (APAFIS N°0300703 and N°10065).

## Results

### Anti-hPAD4 Antibodies in Mice Immunized With hPAD4

To check the effectiveness of hPAD4 immunization, we analyzed IgG responses to hPAD4 by ELISA in 20 mice immunized with hPAD4 and 21 mice immunized with PBS. Sera collected 15, 30, 45 and 60 days post first immunization were used for anti-hPAD4 IgG antibody detection (Immunization protocol and antibody analysis kinetic detailed in **Supplementary Figure 3**).

**Figure 1:**
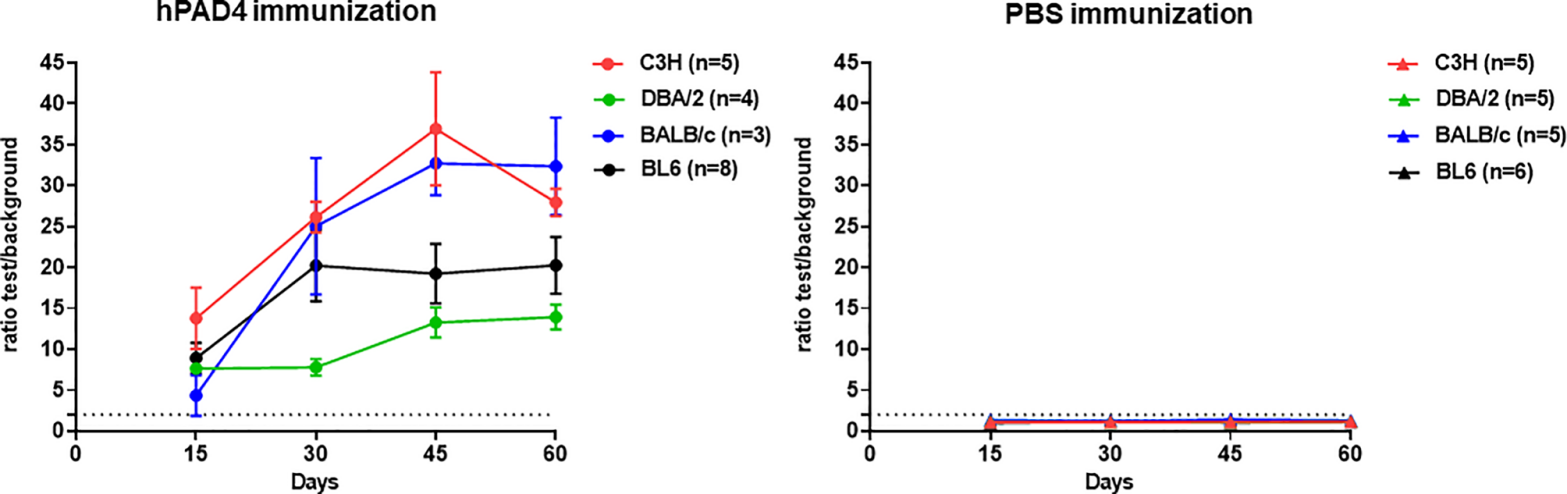
IgG response to hPAD4. Anti-hPAD4 IgG was evaluated by ELISA using sera obtained at 15, 30, 45 and 60 days post first immunization. Background OD was obtained by adding each serum to a well without protein. Sera were evaluated by measuring the ratio test OD/background OD. Ratios equal or higher than 2 defined positive sera. Means +/- SEM were calculated for each group of mice.

All the hPAD4 immunized mice developed anti-hPAD4 IgG while none of the PBS immunized mice did (Fisher exact test, p=4×10^−12^) (**Figure 1**).

### Anti-Citrullinated Peptide Autoantibody Pattern in Mice Immunized With hPAD4

Sera from 20 mice immunized with hPAD4 and 21 mice immunized with PBS were used to probe arrays containing 33 arginine peptides and their 136 citrulline-substituted variants. Each peptide was tested in triplicate. The presence of peptide specific IgG antibodies was detected with a fluorescently labelled anti-mouse IgG antibody. For each strain, background mean signal intensity was obtained for each peptide using sera from mice immunized with PBS. A positive serum was defined by a ratio (mean test signal/mean background signal) higher than 3.

**Figure 2:**
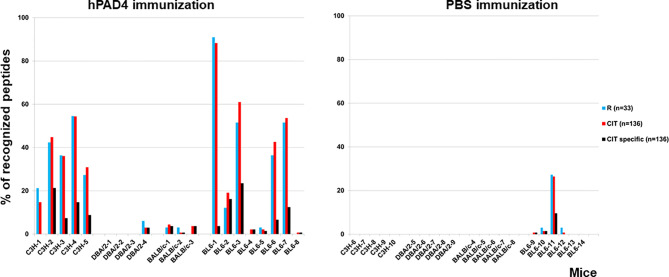
Percentage of recognized peptides in C3H, DBA/2, BALB/c and BL6 mice immunized with hPAD4 or PBS. Peptide arrays made of 33 arginine-containing peptides and their 136 citrullinated variants were used to screen the sera of mice immunized with hPAD4 or PBS. For each strain, background mean signal intensity was obtained for each peptide using sera from mice immunized with PBS. A positive serum was defined by a ratio (mean test signal/mean background signal) higher than 3.

**Figure 3:**
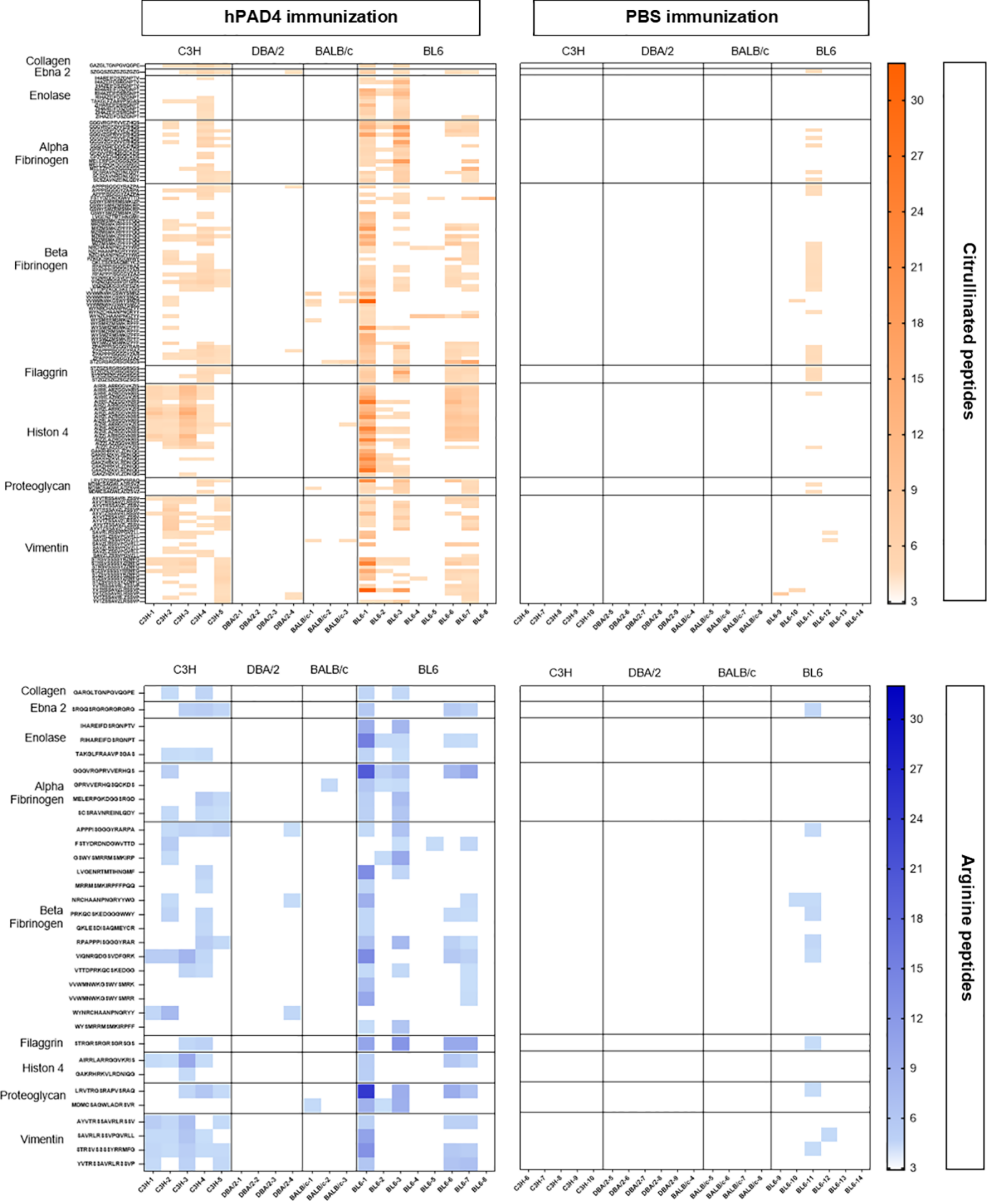
Heatmap of anti-arginine and citrullinated IgG responses in mice immunized with hPAD4 or PBS. A heatmap diagram was computed with ratios (mean test signal/mean background signal) in a color-coded manner from white (no binding) to red (strong binding) for citrullinated peptides and from white (no binding) to blue (strong binding) for arginine peptides. X-axis represents mice (on the left, mice immunized with hPAD4, on the right, mice immunized with PBS), Y-axis represents peptides.

IgG anti-citrullinated peptides antibodies were detected in 17/20 (85%) mice immunized with hPAD4 versus 4/21 (19%) mice immunized with PBS (Fisher exact test, p=3×10^−5^) (**Figures 2**, **3**).

**Figure 4:**
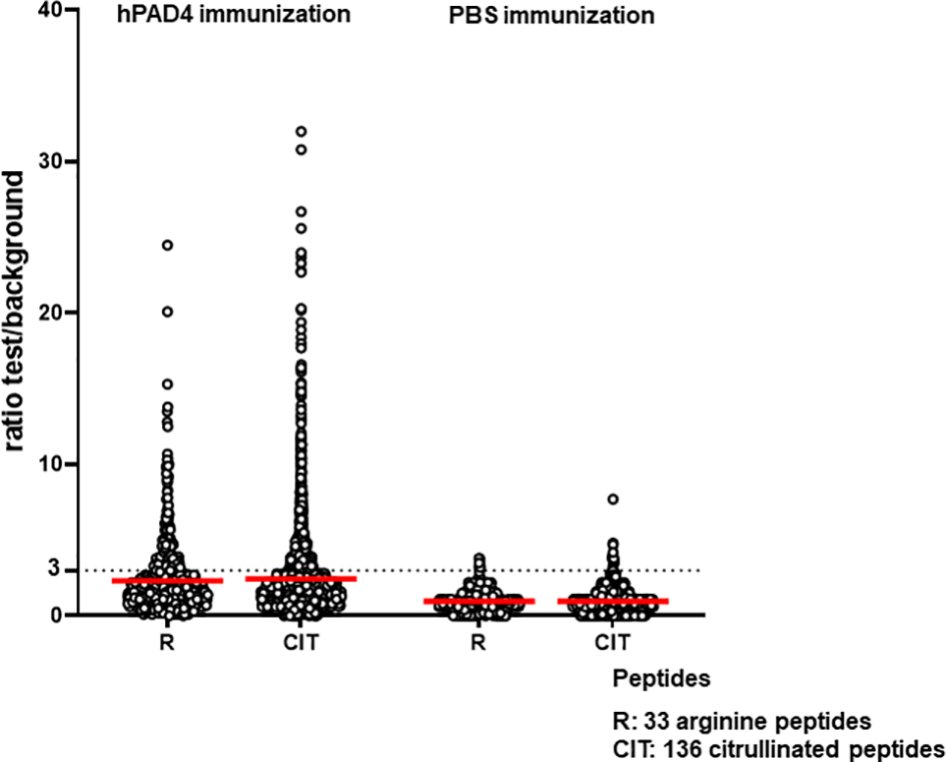
Anti-arginine and citrullinated IgG responses in mice immunized with hPAD4 (n=20) or PBS (n=21). A ratio test/background was calculated for each arginine (R) and citrulinated peptide (CIT). A positive serum was defined by a ratio higher than 3.

Anti-citrullinated peptide response was higher in mice immunized with hPAD4 than in mice immunized with PBS (Mann Whitney test, p<0.0001) (**Figure 4**).

Antibodies induced by hPAD4 immunization were not specific for citrullinated peptides. Indeed, IgG antibodies to arginine peptides were also detected in 14/20 (70%) mice immunized with hPAD4 versus 3/21 (14%) mice immunized with PBS (Fisher exact test, p= 0.0004) (**Figure 2**). Anti-arginine peptide IgG response was higher in mice immunized with hPAD4 than in mice immunized with PBS (Mann Whitney test, p<0.0001) (**Figure 4**). Average ratio was 2.4 (+/- 3 SD) for anti-citrullinated peptides against 2.3 (+/- 2.4 SD) for anti-arginine peptides (Mann Whitney test, p=0.26) (**Figure 4**).

### Influence of H-2 Haplotypes on PAD Induced Production of ACPAs

To test whether MHC class II polymorphism influences ACPA production, we compared anti-citrullinated peptide antibody patterns in DBA/2 (H-2d), BALB/c (H-2d), BL6 (H-2b) and C3H (H-2k) mice immunized with hPAD4.

**Figure 5:**
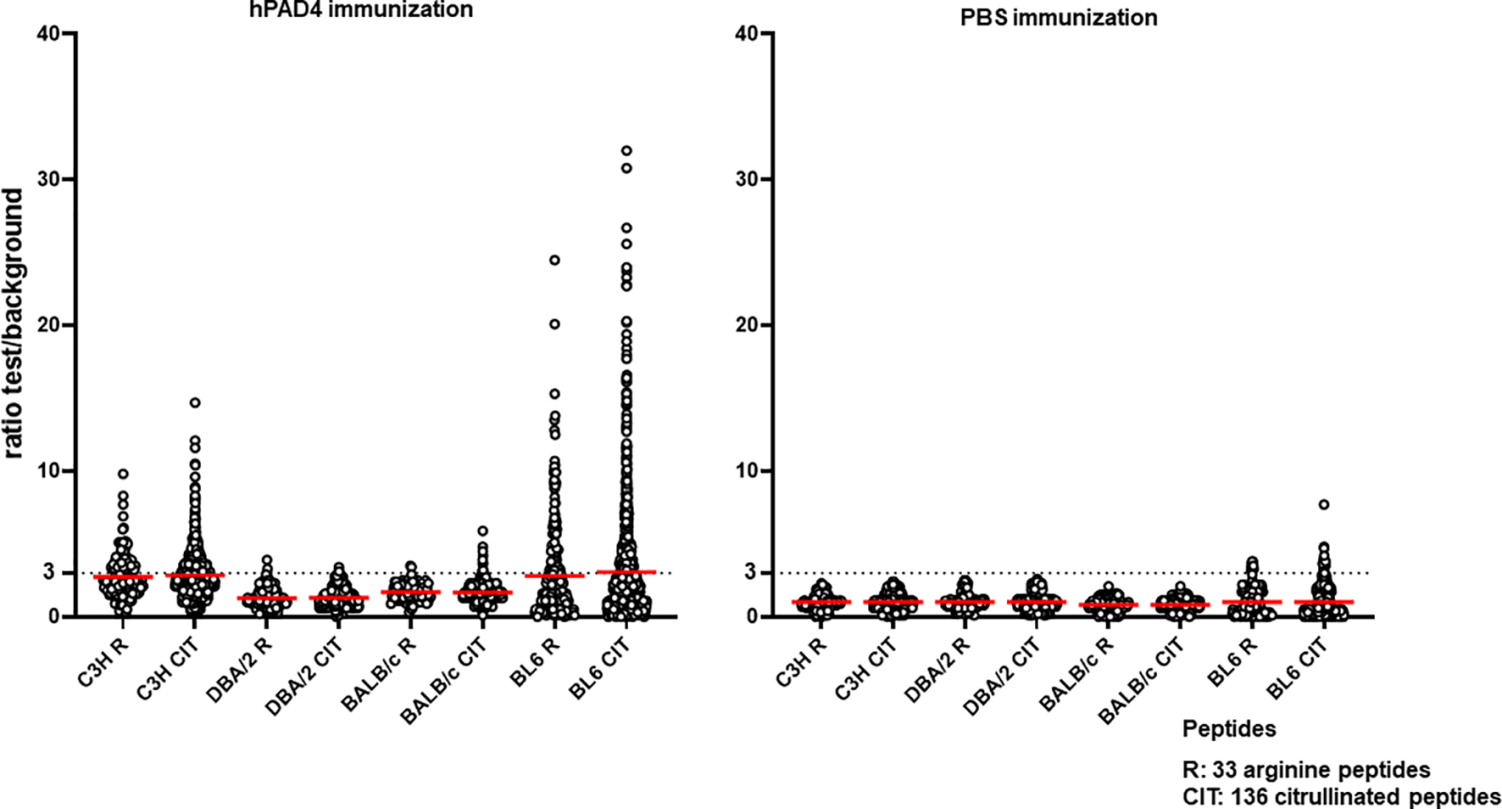
Anti-arginine and citrullinated IgG responses in each mouse strain immunized with hPAD4 or PBS. A ratio test/background was calculated for each arginine (R) and citrulinated peptide (CIT). A positive serum was defined by a ratio higher than 3.

DBA/2 and BALB/c mice (H-2d) displayed weak anti-citrullinated peptide IgG responses (**Figures 2**, **3**, **5**).

IgG antibodies to citrullinated or arginine peptides were detected in 1/4 (25%) DBA/2 mice immunized with hPAD4 versus 0/5 DBA/2 mice immunized with PBS (Fisher’s exact test, p= 0.44) (**Figure 2**). Average ratio was 1.3 (+/- 0.6 SD) for anti-citrullinated peptides IgG and 1.3 (+/- 0.8 SD) for anti-arginine peptides IgG (Mann Whitney test, p=0.8) (**Figure 5**).

IgG antibodies to citrullinated peptides were detected in 3/3 (100%) BALB/c mice immunized with hPAD4 versus 0/5 BALB/c mice immunized with PBS (Fisher’s exact test, p= 0.02) (**Figure 2**). IgG antibodies to arginine peptides were detected in 2/3 (66%) BALB/c mice immunized with hPAD4 versus 0/5 BALB/c mice immunized with PBS (Fisher’s exact test, p= 0.1) (**Figure 2**). Average ratio was 1.7 (+/- 0.6 SD) for anti-citrullinated peptides IgG and 1.7 (+/- 0.6 SD) for anti-arginine peptides IgG (Mann Whitney test, p=0.3) (**Figure 5**).

BL6 (H-2b) and C3H mice (H-2k) displayed strong anti-citrullinated peptide IgG responses (**Figures 2**, **3**, **5**).

IgG antibodies to citrullinated peptides were detected in 8/8 (100%) BL6 mice immunized with hPAD4 versus 4/6 (67%) BL6 mice immunized with PBS (Fisher’s exact test, p= 0.16) (**Figure 2**). IgG antibodies to arginine peptides were detected in 6/8 (75%) BL6 mice immunized with hPAD4 versus 3/6 (50%) BL6 mice immunized with PBS (Fisher’s exact test, p= 0.6) (**Figure 2**).

After hPAD4 immunization, average ratio was 3.1 (+/- 4.4 SD) for anti-citrullinated peptides IgG and 2.8 (+/- 3.4 SD) for anti-arginine peptides IgG (Mann Whitney test, p= 0.11) (**Figure 5**).

After PBS immunization, average ratio was 1 (+/- 1.1 SD) for anti-citrullinated peptides IgG and 1 (+/- 1 SD) for anti-arginine peptides IgG (Mann Whitney test, p= 0.9) (**Figure 5**).

IgG antibodies to citrullinated or arginine peptides were detected in 5/5 (100%) C3H mice immunized with hPAD4 versus 0/5 C3H mice immunized with PBS (Fisher exact test, p= 0.008) (**Figure 2**). Average ratio was 2.8 (+/- 1.7 SD) for anti-citrullinated peptides IgG and 2.7 (+/-1.5 SD) for anti-arginine peptides IgG (Mann Whitney test, p=0.6) (**Figure 5**).

Major peptide epitopes recognized by at least 60% of mice immunized with hPAD4 and not by PBS were detailed in **Supplementary Figure 4**.

### Autoantibody Pattern Specific for Citrullinated Peptides After hPAD4 Immunization

**Figure 6:**
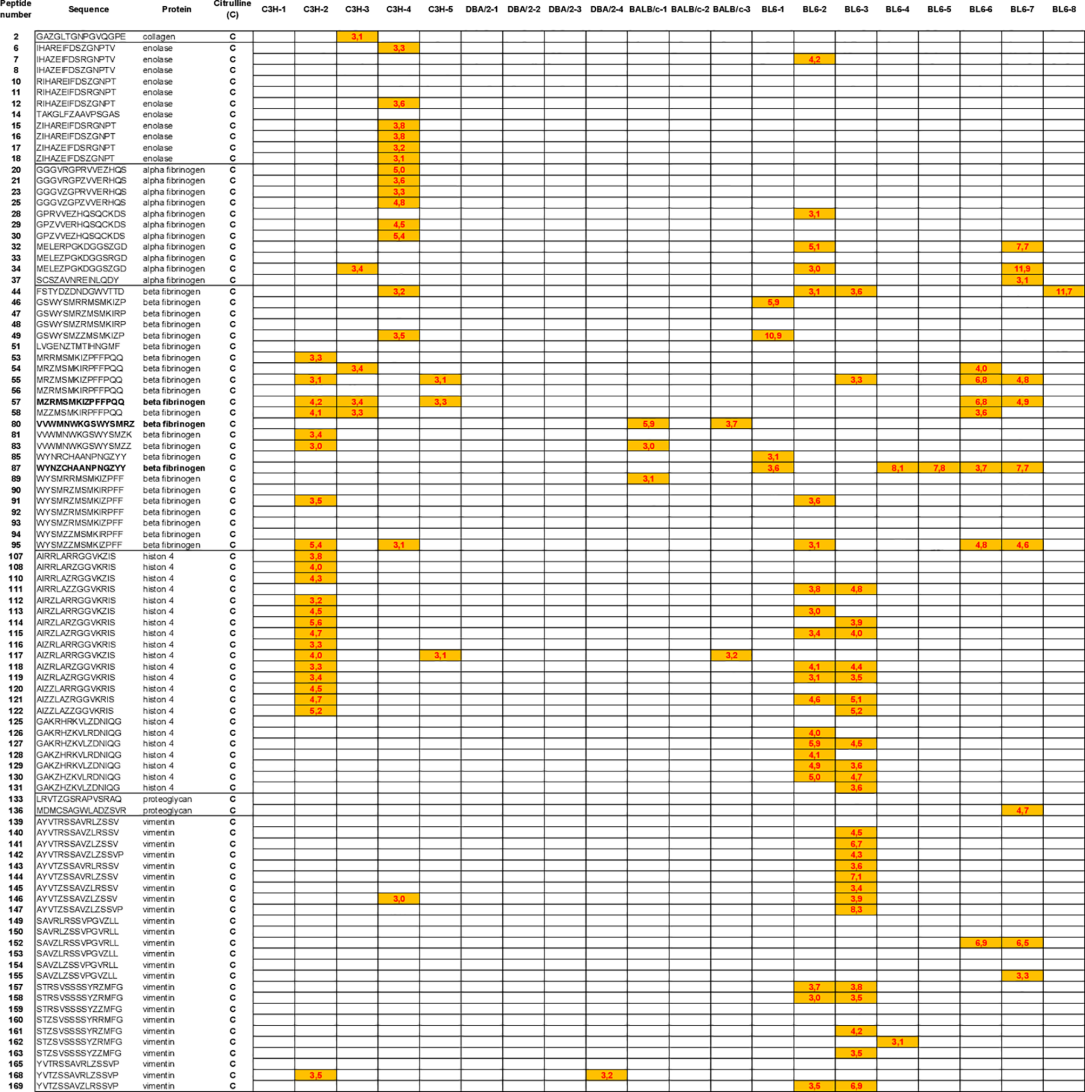
Autoantibody pattern specific for citrullinated peptides after hPAD4 immunization. Peptides recognized by hPAD4 immunized mice and no PBS immunized mice were identified. A ratio test/background was calculated for each arginine and citrulinated peptide. Specific IgG response to citrullinated peptide was defined by a ratio higher than 3 for citrullinated peptide and by a ratio lower than 3 for its arginine-substituted variant (orange rectancle).

We have identified the peptides only recognized in their citrulline forms (**Figure 6**). To identify major citrullinated peptides, we arbitrarily selected those recognized by at least 60% of mice immunized with hPAD4 and not by PBS immunized mice.

No major citrullinated peptides were recognized by DBA/2 mice. Three peptides of the beta fibrinogen were specifically recognized in their citrullinated forms, the peptides 80, 87 and 57 by respectively, 66% of BALB/c, 62% of BL6 and 60% of C3H mice.

## Discussion

The sera of two thirds of patients with RA contain disease-specific autoantibodies, namely IgG ACPA. ACPA recognize citrullinated epitopes on multiple proteins. Citrulline is generated by post translational conversion of arginine by enzymes called PADs. The mechanisms leading to the production of ACPAs are unknown.

The association between RA and particular HLA-DRB1 alleles suggests that HLA-DR restricted T cells help antibody responses to the very numerous citrullinated proteins known to be recognized by ACPAs. However, the identity of the peptides whose presentation by RA associated HLA-DR molecules and recognition by helper T cells provides help to the B cells specific for the many citrullinated antigens recognized by RA patients’ ACPAs is still unknown. Indeed, approximately 100.000 distinct citrullinated peptides are recognized by RA specific autoantibodies, suggesting a requirement for many helper T lymphocytes with different specificities (10).

In 2003, Hill et al. proposed that shared epitope positive RA associated HLA-DR molecules bound citrullinated peptides with high affinity because their basic P4 pocket allowed them bind citrullinated peptides (neutral) better than arginin peptides (basic). Hill and Cairns, in collaboration with the Sette group, studied the binding affinity of a peptide from vimentin called Vim 65-77 and its citrullinated variant, called Vim R70Cit for purified HLA-DR molecules encoded by HLA-DRB1*01:01, HLA-DRB1*04:01, HLA-DRB1*04:04, HLA-DRB1*03:01, HLA-DRB1*07:01, HLA-DRB1*08:02, HLA-DRB1*11:01, HLA-DRB1*13:01. For the first three HLA-DR molecules, containing the shared epitope and associated with RA, the binding affinity for Vim R70Cit was higher than for Vim 65-77 (11).

In this seminal article, the binding affinity of Vim 65-77 and Vim R70-Cit was calculated for 8 HLA-DR alleles, not including HLA-DRB1*04:02, an HLA-DR4 subtype which is not associated with RA. No other peptide was included in this study.

This article launched the hypothesis that shared epitope positive HLA-DR alleles present citrullinated peptides to the T cells that help the development of ACPA.

Following on this hypothesis, Hill and Cairns developed a mouse model of arthritis by immunizing HLA-DRB1*0401 transgenic mice with citrullinated fibrinogen (12). In this type of model as in a more recent model using immunization with citrullinated or homocitrullinated peptides, ACPA develop after citrullinated peptide/protein immunization, thanks to the presentation of citrullinated peptides to helper T cells (13).

In 2013, Scally et al. performed cristallographic studies of HLA-DRB1*04:01 HLA-DRB1*04:04, HLA-DRB1*04:02. They showed the structure of Vim R70Cit bound to HLA-DRB1*04:01, *04:04 and HLA-DRB1*04:02, the latter unexpected since HLA-DRB1*04:02 does not contain the shared epitope and is not associated with RA (14).

Finally, in 2017, The Sette team revisited their 2003 binding affinity study, this time using 200 peptides (including vim 65-77) and 28 purified HLA-DR molecules.

Their conclusion was that “RA associated epitopes in their wild type and citrullinated forms have variable binding patterns to HLA class II alleles and a consistent impact of citrullination is not apparent” (15).

Because our extensive HLA-DR fibrinogen peptides binding data did not indicate preferential binding of citrullinated peptides to RA associated HLA-DR molecules (16), we looked for an alternative mechanism and proposed that PADs might be the T cell targets whose recognition provides help for the production of IgG ACPA by a classical hapten carrier model where PADs are the carriers and PAD-bound peptides are the haptens (7).

To demonstrate this point, we developed a mouse model by immunizing normal mice with human or murine PADs.

C3H mice, which express an I-E beta k molecule whose amino acid sequence is very similar to that of RA associated HLA-DRβ1*04:01, develop IgG anti-citrullinated fibrinogen antibodies after PAD4 or PAD2 immunization (8). In contrast, DBA/2 mice, which express an I-E beta d chain whose amino acid sequence is very similar to that of “non-RA associated” HLA-DRβ1*04:02, failed to develop antibodies to citrullinated fibrinogen peptides.

In this early study, ACPA production was detected in 30% of C3H mice immunized with hPAD4 by ELISA on fifty fibrinogen peptides.

Here, we developed a more sensitive assay to analyze ACPAs in mice after PAD immunization. We designed peptide arrays containing 33 arginine-containing peptides and their 136 citrulline-substituted variants. We chose 15 mers from antigens (collagen, filaggrin, ebna, proteoglycan, enolase, alpha and beta fibrinogen, histon 4 and vimentin) known to be recognized by human ACPAs (17–28). Peptides were centered on Cit-Gly motifs known to optimize the binding of ACPAs (29). Peptide arrays were used to screen the sera of C3H, DBA/2, BALB/c, BL6 mice immunized with hPAD4 or PBS to test the influence of H-2 haplotypes on ACPAs induced by hPAD4 immunization.

We confirmed that hPAD4 immunization induces IgG ACPA in mice. Indeed, 85% of the mice immunized with hPAD4 developed IgG ACPA.

IgG ACPA induced by hPAD4 immunization are not specific for citrullinated peptides. Indeed, most peptides are recognized under their arginine and citrulline forms.

This is expected in our hapten carrier model: helper T cells which recognize peptides from hPAD will help B cells specific for peptides bound by hPAD4 whether or not they have been citrullinated. What makes the carrier effect possible is the contact between PAD and its substrate(s), because it allows processing of the carrier which binds the substrate and contributes a peptide which activates helper T cells.

In this respect, we noticed that 3% of our RA patients with a positive anti-ccp2 test also have a positive anti-cap test (the equivalent test using a cyclic arginine peptide rather than a cyclic citrullinated peptide) (data not shown).

We observed that the ACPA response induced by hPAD4 immunization is not directed against peptides from one but from many proteins (alpha and beta fibrinogen, histon 4, vimentin). This is similar to what occurs in humans, where ACPAs recognize numerous citrullinated proteins (10), which fits the hapten/carrier model (7).

DBA/2 and BALB/c mice (H-2d) have the lowest anti-citrullinated peptide IgG responses. This confirms our previous study in DBA/2 mice (8).

C3H (H-2k) and BL6 mice (H-2b) have the highest anti-citrullinated peptides IgG responses. However, the ACPA response is heterogeneous in BL6 mice. Some mice develop strong IgG ACPA responses, while others do not develop any. BL6 mice (H-2b) do not express I-E, because the I-E alpha chain is not functional on the H-2b haplotype. Thus, I-Ab is the only class II MHC molecule available to BL/6 mice to develop their immune system.

It has been shown that peptides from PADs presented by I-Ab are involved in the selection of the T cell repertoire in C57BL/6 mice (30). A temporal thymic selection switch and ligand binding kinetics constrain neonatal Foxp3+ Treg cell development (30). This might explain why in our hPAD4 immunization model, BL6 mice develop T cells specific for PAD4, and ACPA. This is different from the arthritis induced by citrullinated fibrinogen immunization in BL6, DR4 transgenic, I-A knock out model which relies on DR4 binding of citrullinated peptides to activate citrullinated peptide specific B cells (12).

In this study, we confirmed that C3H mice which express an I-E beta k chain homologous to the RA associated HLA-DRB1*04:01 allele display the best anti-citrullinated protein antibody response after PAD4 immunization.

In conclusion, we have developed a sensitive peptide array, capable to detect ACPAs in 100% of C3H mice immunized with hPAD4. To our knowledge no custom or commercialized assays are available to measure murine ACPA response. This peptide array will be a critical tool to monitor the effect of PAD tolerization on ACPA production in C3H mice previously immunized with PAD.

## Data Availability Statement

The original contributions presented in the study are included in the article/**Supplementary Material**. Further inquiries can be directed to the corresponding author.

## Ethics Statement

The animal study was reviewed and approved by Animal Ethics Committee of Marseille and the Ministère de l’Enseignement Supérieur et de la Recherche, France (APAFIS N°0300703 and N°10065).

## Author Contributions

JR and IA designed research. MH, NL, FA, and IA performed research. MH, NL, IA, and JR analyzed data. JR and IA wrote the paper. All authors contributed to the article and approved the submitted version.

## Conflict of Interest

The authors declare that the research was conducted in the absence of any commercial or financial relationships that could be construed as a potential conflict of interest.

## Publisher’s Note

All claims expressed in this article are solely those of the authors and do not necessarily represent those of their affiliated organizations, or those of the publisher, the editors and the reviewers. Any product that may be evaluated in this article, or claim that may be made by its manufacturer, is not guaranteed or endorsed by the publisher.
