# Supplementary material for: PAD4 Immunization Triggers Anti-Citrullinated Peptide Antibodies in Normal Mice: Analysis With Peptide Arrays
Source: Front Immunol. 2022 Mar 31;13:840035. doi: 10.3389/fimmu.2022.840035 (PMC9008206; doi:10.3389/fimmu.2022.840035)
Supplement: Supplementary file 1 [file Image_1.pdf]

Supplementary figure 1: Ratios for mice immunized with hPAD4

hPAD4 immunization

Peptide number	Sequence	Protein	Arginine (R) citrulline (C)	C3H-1	C3H-2	C3H-3	C3H-4	C3H-5	DBA/2-1	DBA/2-2	DBA/2-3	DBA/2-4	BALB/c-1	BALB/c-2	BALB/c-3	BL6-1	BL6-2	BL6-3	BL6-4	BL6-5	BL6-6	BL6-7	BL6-8
1	GARGLTGNPGVQGPE	collagen	R	1,5	3,3	2,9	4,1	2,0	0,7	0,6	1,3	1,9	1,7	1,3	2,3	4,0	2,1	3,7	0,7	0,7	1,4	1,7	0,3
2	GAZGLTGNPGVQGPE	collagen	C	1,3	3,1	3,1	5,7	3,0	0,7	0,7	1,5	2,1	1,6	1,2	2,1	5,3	2,5	4,5	0,6	1,1	2,1	2,4	0,3
3	SRGQSRGRGRGRGRG	ebna 2	R	2,9	2,5	4,8	5,1	4,0	0,4	1,0	1,5	2,6	1,5	1,2	2,3	5,7	1,3	2,6	1,6	1,3	5,6	4,4	0,9
4	SZGQSZGZGZGZGZG	ebna 2	C	1,1	2,4	3,0	5,9	3,9	0,7	0,7	1,1	3,2	1,6	1,2	1,9	6,9	1,8	4,3	0,5	1,3	4,2	3,0	0,5
5	IHAREIFDSRGNPTV	enolase	R	0,3	2,1	0,8	2,2	1,8	2,2	0,8	1,6	1,1	1,5	1,1	1,4	9,2	2,6	8,2	0,2	0,4	1,3	1,3	0,1
6	IHAREIFDSZGNPTV	enolase	C	0,5	2,1	1,2	3,3	2,2	1,3	0,7	1,6	1,4	1,4	1,1	1,4	4,7	2,2	5,3	0,3	0,6	1,3	2,0	0,2
7	IHAZEIFDSRGNPTV	enolase	C	0,6	2,3	1,2	2,5	1,9	1,3	0,6	1,4	1,1	2,2	1,6	2,3	3,4	4,2	11,3	0,3	0,6	0,9	2,4	0,3
8	IHAZEIFDSZGNPTV	enolase	C	0,7	2,3	1,4	2,5	2,2	1,7	0,6	1,2	1,6	1,5	1,1	1,6	2,2	1,9	4,8	0,4	0,9	0,6	2,9	0,6
9	RIHAREIFDSRGNPT	enolase	R	0,9	2,4	1,5	2,7	2,1	1,6	0,9	2,1	1,5	0,9	1,9	1,5	15,3	3,4	3,4	0,5	1,0	3,3	3,2	0,5
10	RIHAREIFDSZGNPT	enolase	C	0,4	1,7	0,9	2,5	1,4	1,7	0,9	1,5	1,4	1,3	1,2	1,5	13,1	3,0	8,8	0,3	0,4	1,6	1,9	0,1
11	RIHAZEIFDSRGNPT	enolase	C	0,5	2,3	0,9	2,9	1,6	1,6	0,6	1,5	1,4	1,7	1,6	2,3	10,5	4,3	11,3	0,2	0,5	1,4	2,0	0,1
12	RIHAZEIFDSZGNPT	enolase	C	0,7	2,1	1,4	3,6	2,2	1,1	0,7	1,2	1,5	1,6	1,3	1,8	6,8	2,9	7,1	0,3	0,6	0,9	2,0	1,1
13	TAKGLFRAAVPSGAS	enolase	R	1,2	3,2	3,0	3,3	1,8	0,8	0,7	1,2	1,1	1,1	1,1	1,5	4,2	1,5	3,0	0,3	0,3	1,2	0,8	0,1
14	TAKGLFZAAVPSGAS	enolase	C	1,3	3,0	3,0	3,0	1,9	0,8	0,8	0,8	1,5	1,5	1,1	1,9	3,7	2,6	3,7	0,4	0,9	1,3	2,1	0,1
15	ZIHAREIFDSRGNPT	enolase	C	0,6	2,8	1,3	3,8	2,2	2,1	0,8	1,6	2,0	1,3	1,3	1,5	10,8	2,5	4,2	0,3	0,9	2,6	2,0	0,4
16	ZIHAREIFDSZGNPT	enolase	C	0,5	1,7	1,2	3,8	2,1	1,5	0,8	1,3	1,9	1,6	1,3	1,7	5,9	1,8	5,2	0,4	0,6	2,0	2,4	0,2
17	ZIHAZEIFDSRGNPT	enolase	C	0,7	2,3	1,3	3,2	2,0	1,6	0,7	1,3	1,8	1,5	1,3	1,8	6,3	2,1	6,3	0,3	0,3	1,6	2,4	0,1
18	ZIHAZEIFDSZGNPT	enolase	C	0,8	1,8	1,5	3,1	2,1	1,0	0,6	1,1	1,6	1,4	1,2	1,6	3,2	1,8	5,4	0,7	1,1	1,9	3,5	1,2
19	GGGVRGPRVVERHQS	alpha fibrinogen	R	1,5	4,7	2,2	2,5	1,6	1,5	1,0	1,8	1,8	1,1	1,3	1,8	20,1	4,7	6,5	1,0	2,5	7,8	10,3	0,5
20	GGGVRGPRVVEZHQS	alpha fibrinogen	C	0,5	3,9	1,2	5,0	2,7	1,3	0,9	1,4	1,5	0,8	1,2	2,1	10,1	3,0	6,2	0,5	1,0	4,1	4,5	0,3
21	GGGVRGPZVVERHQS	alpha fibrinogen	C	0,5	3,9	1,1	3,6	2,0	1,0	0,6	1,4	2,0	1,1	1,2	1,9	12,9	4,9	18,9	1,2	1,2	4,4	5,9	0,5
22	GGGVRGPZVVEZHQS	alpha fibrinogen	C	0,8	2,0	2,1	5,3	3,5	1,0	0,6	1,2	2,3	2,2	1,8	2,6	6,7	0,1	9,2	1,0	0,9	7,2	5,9	0,2
23	GGGVZGPRVVERHQS	alpha fibrinogen	C	0,6	3,2	1,1	3,3	1,8	1,4	0,7	1,4	1,0	1,4	1,4	1,4	16,1	3,1	16,4	0,9	1,3	4,3	4,2	0,3
24	GGGVZGPRVVEZHQS	alpha fibrinogen	C	0,7	2,0	1,7	6,2	3,6	1,0	0,6	1,2	2,2	1,2	1,2	1,5	4,5	1,2	3,8	0,5	0,9	2,0	2,4	0,4
25	GGGVZGPZVVERHQS	alpha fibrinogen	C	1,0	3,3	2,1	4,8	2,7	0,8	0,5	1,0	1,8	1,9	1,3	1,8	5,1	2,0	16,1	0,4	0,9	1,5	2,8	0,6
26	GGGVZGPZVVEZHQS	alpha fibrinogen	C	0,8	2,3	2,0	5,8	3,2	1,1	0,6	1,2	2,1	1,9	1,8	2,7	4,0	0,9	8,5	0,7	1,3	2,4	4,7	0,2
27	GPRVVERHQSQCKDS	alpha fibrinogen	R	0,5	2,0	1,7	1,9	1,5	1,5	0,3	1,0	0,9	1,5	3,4	2,6	6,0	3,0	3,3	0,3	0,4	0,5	0,7	0,2
28	GPRVVEZHQSQCKDS	alpha fibrinogen	C	1,1	2,5	2,5	2,4	1,9	0,9	0,6	1,0	1,1	1,4	1,2	2,3	5,0	3,1	4,9	0,2	0,5	0,8	1,0	0,2
29	GPZVVERHQSQCKDS	alpha fibrinogen	C	1,1	2,7	2,4	4,5	1,8	0,9	0,6	1,2	1,4	1,9	1,5	2,4	4,6	1,6	6,3	0,6	0,5	1,0	2,1	0,2
30	GPZVVEZHQSQCKDS	alpha fibrinogen	C	1,1	1,9	2,4	5,4	2,9	1,0	1,1	1,2	2,4	1,9	1,2	1,8	3,9	2,6	4,4	0,5	0,8	1,4	2,2	0,3
31	MELERPGKDGGSRGD	alpha fibrinogen	R	1,0	2,2	2,1	5,1	3,7	1,1	0,6	1,2	2,4	1,3	1,0	1,6	4,8	2,6	7,3	0,5	2,4	2,2	2,8	0,4
32	MELERPGKDGGSZGD	alpha fibrinogen	C	1,0	2,3	2,3	2,7	1,8	1,2	0,7	1,3	2,2	2,1	1,5	2,1	4,4	5,1	18,0	0,3	1,0	2,0	7,7	0,3
33	MELEZPGKDGGSRGD	alpha fibrinogen	C	1,3	2,2	2,6	5,3	2,8	1,1	0,7	1,2	2,2	1,5	1,3	1,7	4,5	2,8	6,8	0,6	1,3	2,0	2,7	0,5
34	MELEZPGKDGGSZGD	alpha fibrinogen	C	1,1	2,3	3,4	3,7	2,5	1,3	0,7	1,2	2,3	2,5	1,9	2,7	2,4	3,0	5,3	1,1	1,3	2,3	11,9	0,3
35	SCSRAVNREINLQDY	alpha fibrinogen	R	0,5	3,5	1,1	3,5	3,1	1,0	0,5	1,1	2,4	1,6	1,0	1,4	4,6	2,3	6,1	0,9	1,0	1,3	1,9	0,4
36	SCSRAVNZEINLQDY	alpha fibrinogen	C	0,8	2,2	1,7	4,3	3,5	0,9	0,6	1,2	2,7	1,7	1,3	1,7	5,8	0,7	6,7	0,7	0,9	4,1	3,2	0,2
37	SCSZAVNREINLQDY	alpha fibrinogen	C	0,8	1,8	1,8	3,5	3,0	1,2	0,6	1,2	1,9	2,5	2,0	2,6	3,2	1,0	6,2	0,2	0,4	1,5	3,1	0,1
38	SCSZAVNZEINLQDY	alpha fibrinogen	C	0,9	2,3	1,9	4,0	3,0	1,1	0,7	1,3	2,4	1,6	1,3	2,0	5,5	1,2	6,7	0,4	0,7	2,9	3,5	0,2
39	APPPISGGGYRARPA	beta fibrinogen	R	1,8	3,3	4,2	4,0	4,6	0,6	0,7	1,5	3,0	1,4	2,2	2,1	3,5	0,8	6,5	0,4	0,5	1,4	1,4	0,3
40	APPPISGGGYRAZPA	beta fibrinogen	C	0,9	1,9	2,0	4,7	3,2	1,0	0,9	1,3	3,4	1,8	1,4	2,2	6,8	1,3	3,1	0,6	0,7	2,7	2,0	0,3
41	APPPISGGGYZARPA	beta fibrinogen	C	2,1	3,8	4,4	4,5	3,2	0,5	0,8	1,6	2,5	1,8	1,4	2,2	2,6	0,9	1,7	0,5	0,7	1,6	1,7	0,4
42	APPPISGGGYZAZPA	beta fibrinogen	C	1,0	2,3	2,8	5,0	2,9	1,0	0,7	1,2	2,8	1,6	1,1	1,9	4,4	1,7	3,4	0,6	1,0	2,8	2,2	0,3
43	FSTYDRDNDGWVTTD	beta fibrinogen	R	0,9	5,2	1,7	2,8	1,8	0,7	0,4	1,0	2,6	1,8	1,1	1,7	2,3	1,6	3,0	2,2	3,2	1,6	3,8	1,4
44	FSTYDZDNDGWVTTD	beta fibrinogen	C	1,0	5,4	2,3	3,2	2,0	1,0	0,5	1,0	2,7	2,1	1,3	1,3	2,8	3,1	3,6	2,4	3,5	1,9	5,8	11,7
45	GSWYSMRRMSMKIRP	beta fibrinogen	R	1,4	3,2	1,8	1,5	2,3	1,5	0,5	1,5	2,4	2,5	2,6	2,3	2,9	3,3	10,0	0,4	0,3	0,6	1,1	0,5
46	GSWYSMRRMSMKIZP	beta fibrinogen	C	0,9	2,4	2,0	2,0	2,0	1,0	0,5	2,1	2,1	1,8	1,8	1,4	5,9	1,6	4,5	0,6	0,3	0,4	0,8	0,2
47	GSWYSMRZMSMKIRP	beta fibrinogen	C	1,1	2,2	2,9	1,7	2,5	0,7	0,5	1,8	2,5	2,1	1,7	1,6	2,7	1,1	1,7	0,3	0,3	0,4	0,4	0,3
48	GSWYSMZRMSMKIRP	beta fibrinogen	C	1,0	2,0	1,9	1,8	2,7	1,0	0,8	1,5	2,5	1,4	1,7	1,4	2,7	0,7	1,8	0,3	0,3	0,3	0,4	0,3
49	GSWYSMZZMSMKIZP	beta fibrinogen	C	0,6	2,3	1,1	3,5	2,3	0,4	0,5	0,9	1,2	2,8	1,4	1,0	10,9	2,0	4,4	0,7	0,7	2,1	1,5	0,0
50	LVGENRTMTIHNGMF	beta fibrinogen	R	0,4	2,0	1,0	4,1	2,2	2,7	0,8	1,4	0,6	1,3	2,3	2,0	13,5	2,3	4,0	0,4	0,2	2,3	1,0	0,2
51	LVGENZTMTIHNGMF	beta fibrinogen	C	0,5	2,4	1,3	3,9	2,2	2,6	0,7	1,5	1,1	1,9	1,5	1,8	9,5	2,8	5,7	0,5	0,4	1,2	1,3	0,3
52	MRRMSMKIRPFFPQQ	beta fibrinogen	R	1,2	2,5	2,2	3,2	2,6	0,8	0,7	1,4	2,3	2,0	1,3	1,8	4,1	1,3	1,0	0,8	0,9	1,8	2,2	0,9
53	MRRMSMKIZPFFPQQ	beta fibrinogen	C	2,4	3,3	2,9	2,6	2,2	0,8	1,1	1,4	1,8	1,3	0,7	1,2	7,6	2,0	1,6	1,0	1,2	2,6	2,8	0,8
54	MRZMSMKIRPFFPQQ	beta fibrinogen	C	2,5	3,0	3,4	2,8	2,1	0,5	1,3	1,4	2,0	1,8	0,6	1,3	11,8	2,6	2,2	1,3	1,5	4,0	3,0	0,7
55	MRZMSMKIZPFFPQQ	beta fibrinogen	C	1,6	3,1	2,1	5,1	3,1	0,2	0,7	0,8	2,1	1,3	0,4	1,2	18,4	2,6	3,3	1,7	2,1	6,8	4,8	0,8
56	MZRMSMKIRPFFPQQ	beta fibrinogen	C	1,6	2,8	2,8	3,0	2,5	0,8	0,9	1,3	2,0	1,9	0,9	2,1	5,1	1,6	1,4	0,9	1,0	2,2	2,3	0,8
57	MZRMSMKIZPFFPQQ	beta fibrinogen	C	2,7	4,2	3,4	6,0	3,3	0,3	0,9	1,2	1,9	1,4	0,6	1,1	14,5	2,9	2,9	1,9	2,3	6,8	4,9	0,4
58	MZZMSMKIRPFFPQQ	beta fibrinogen	C	2,9	4,1	3,3	3,7	2,5	0,7	1,9	1,8	1,8	1,1	0,5	1,0	9,5	2,2	1,5	1,1	1,3	3,6	2,9	0,6

59	MZZMSMKIZPFFPQQ	beta fibrinogen	C	0,7	2,4	1,1	6,1	2,6	0,7	0,6	0,8	2,1	1,2	0,6	0,8	13,3	2,3	3,4	2,0	2,2	8,1	4,8	1,0
60	NRCHAANPNGRYYWG	beta fibrinogen	R	1,7	3,3	1,4	1,6	1,5	1,3	1,0	2,2	3,3	2,5	0,7	2,0	8,9	1,7	1,7	2,3	1,6	1,5	3,4	0,2
61	NRCHAANPNGZYYWG	beta fibrinogen	C	1,2	2,8	1,2	2,5	2,3	0,3	0,7	1,5	2,2	1,9	0,7	1,1	3,1	0,4	0,6	3,7	3,3	1,8	4,3	0,4
62	NZCHAANPNGRYYWG	beta fibrinogen	C	1,5	3,1	1,3	2,8	2,3	0,3	0,8	1,7	2,8	1,9	0,9	1,3	3,6	0,7	0,7	2,7	2,0	2,2	3,3	0,5
63	NZCHAANPNGZYYWG	beta fibrinogen	C	0,9	3,7	1,4	3,4	2,4	0,2	0,4	0,9	1,6	2,8	1,1	1,9	2,7	1,5	2,2	2,7	2,7	2,0	3,7	1,0
64	PRKQCSKEDGGGWWY	beta fibrinogen	R	1,4	4,3	2,1	3,9	2,9	0,6	0,4	1,1	0,9	2,1	0,3	1,4	3,9	1,5	1,6	2,6	2,7	3,4	3,2	1,2
65	PZKQCSKEDGGGWWY	beta fibrinogen	C	1,5	4,8	2,7	3,8	2,6	0,5	0,4	1,2	0,8	2,4	0,3	2,0	3,8	1,6	1,7	0,6	1,8	8,1	3,7	0,3
66	QKLESDISAQMEYCR	beta fibrinogen	R	0,9	2,8	2,6	3,7	1,7	0,9	0,6	1,1	1,5	1,7	1,4	2,2	3,9	1,7	2,9	0,8	1,1	2,3	2,4	0,4
67	QKLESDISAQMEYCZ	beta fibrinogen	C	1,2	3,6	3,1	4,4	1,8	1,0	0,8	1,6	2,2	1,9	1,3	1,9	2,9	1,5	2,5	0,8	1,3	2,6	3,4	0,7
68	RPAPPPISGGGYRAR	beta fibrinogen	R	0,8	2,4	1,8	5,1	3,6	1,1	0,6	1,3	1,5	1,3	1,8	2,4	6,7	1,2	7,8	0,4	0,5	4,6	3,0	0,3
69	RPAPPPISGGGYRAZ	beta fibrinogen	C	0,7	2,1	1,5	4,5	2,7	1,0	0,7	1,2	2,7	1,5	1,2	1,9	5,2	1,1	3,7	0,3	0,7	3,6	2,3	0,3
70	RPAPPPISGGGYZAR	beta fibrinogen	C	0,5	2,3	1,2	5,4	4,1	1,3	0,7	1,2	1,9	1,4	1,2	1,8	5,0	0,9	3,6	0,3	0,7	3,8	2,5	0,3
71	RPAPPPISGGGYZAZ	beta fibrinogen	C	1,8	3,5	4,0	4,7	3,0	0,7	0,8	1,5	2,1	1,5	1,4	2,0	5,4	1,5	2,6	0,6	0,9	3,4	3,3	0,3
72	VIQNRQDGSVDFGRK	beta fibrinogen	R	5,1	5,1	8,3	3,9	2,2	0,3	0,9	1,2	2,9	1,6	0,9	2,4	13,8	1,9	2,4	0,7	1,2	5,7	4,6	0,3
73	VIQNRQDGSVDFGZK	beta fibrinogen	C	1,3	2,8	3,2	5,1	3,5	1,2	0,8	1,4	2,4	1,5	1,3	1,7	6,5	1,8	3,9	0,5	1,2	4,5	3,1	0,5
74	VIQNZQDGSVDFGRK	beta fibrinogen	C	2,8	4,3	5,3	6,4	3,7	0,5	0,7	1,4	2,3	1,7	1,7	1,9	8,8	1,6	3,1	0,7	1,0	4,7	2,6	0,4
75	VIQNZQDGSVDFGZK	beta fibrinogen	C	1,4	2,7	3,3	4,2	3,2	0,9	0,7	1,2	2,6	1,6	1,4	1,9	7,1	1,9	4,8	0,4	1,1	4,0	3,4	0,3
76	VTTDPRKQCSKEDGG	beta fibrinogen	R	1,5	2,5	4,1	3,4	2,4	1,0	0,8	1,3	2,4	1,5	1,3	2,6	3,5	2,6	3,6	0,5	1,1	2,1	3,0	0,3
77	VTTDPZKQCSKEDGG	beta fibrinogen	C	1,5	2,6	3,5	3,6	2,4	0,8	0,6	1,2	2,5	1,7	1,1	2,6	4,2	2,5	4,1	0,9	1,2	2,6	3,8	0,4
78	VVWMNWKGSWYSMRK	beta fibrinogen	R	1,4	2,8	2,0	2,2	1,9	0,5	0,9	1,3	1,9	2,0	0,5	1,8	7,0	0,3	1,2	0,8	0,8	2,6	3,0	0,1
79	VVWMNWKGSWYSMRR	beta fibrinogen	R	1,8	2,3	2,1	2,2	1,9	0,9	1,4	1,7	1,6	1,7	0,7	1,9	10,0	2,0	2,3	0,5	0,7	2,3	3,2	0,4
80	VVWMNWKGSWYSMRZ	beta fibrinogen	C	1,3	2,5	1,1	2,3	1,7	0,9	0,9	1,5	1,2	5,9	0,7	3,7	13,9	0,8	1,8	2,0	1,9	2,3	2,7	0,0
81	VVWMNWKGSWYSMZK	beta fibrinogen	C	1,5	3,4	1,2	2,0	1,7	0,2	0,8	1,3	1,1	2,2	0,3	1,6	5,0	0,7	1,3	1,5	1,6	2,5	2,3	0,1
82	VVWMNWKGSWYSMZR	beta fibrinogen	C	1,8	3,0	1,5	2,2	1,7	1,1	0,9	1,5	1,2	4,8	0,8	4,2	30,8	0,0	2,4	1,2	1,2	1,7	1,4	0,0
83	VVWMNWKGSWYSMZZ	beta fibrinogen	C	0,9	3,0	1,0	2,7	2,1	0,7	0,7	1,3	1,2	3,0	0,8	1,6	5,4	0,2	1,7	1,8	1,6	1,0	2,5	0,0
84	WYNRCHAANPNGRYY	beta fibrinogen	R	3,8	7,7	2,3	1,2	1,6	0,0	0,3	0,0	3,9	1,3	0,0	1,7	0,0	0,0	0,1	0,3	0,2	0,6	1,6	0,1
85	WYNRCHAANPNGZYY	beta fibrinogen	C	1,4	2,7	1,1	1,5	1,9	0,8	0,9	2,0	2,4	1,5	0,6	1,1	3,1	1,9	1,9	1,8	1,5	1,1	2,7	1,1
86	WYNZCHAANPNGRYY	beta fibrinogen	C	1,3	2,7	1,0	1,7	1,9	0,6	0,9	2,1	2,4	1,4	0,7	1,2	4,1	1,8	2,2	1,8	1,3	2,0	3,0	1,4
87	WYNZCHAANPNGZYY	beta fibrinogen	C	1,2	3,4	1,3	1,8	1,9	0,5	0,7	1,4	2,5	2,1	0,9	1,3	3,6	0,6	0,5	8,1	7,8	3,7	7,7	0,5
88	WYSMRRMSMKIRPFF	beta fibrinogen	R	0,9	1,4	1,8	2,5	2,1	0,9	0,6	1,3	2,3	2,5	1,4	2,0	3,2	1,6	6,2	0,4	0,6	1,3	1,2	0,5
89	WYSMRRMSMKIZPFF	beta fibrinogen	C	0,9	1,8	1,8	1,9	1,4	1,4	1,1	1,6	2,0	3,1	2,0	2,4	4,0	0,8	1,4	0,4	0,5	0,9	0,8	0,2
90	WYSMRZMSMKIRPFF	beta fibrinogen	C	1,3	1,9	1,7	1,9	1,6	1,0	0,7	1,0	1,8	1,8	1,2	1,5	3,9	1,0	1,2	0,4	0,6	0,8	0,8	0,2
91	WYSMRZMSMKIZPFF	beta fibrinogen	C	2,2	3,5	2,2	2,2	1,4	0,2	0,9	1,2	2,0	2,0	0,5	1,7	20,2	3,6	4,6	0,7	0,8	2,2	2,0	0,3
92	WYSMZRMSMKIRPFF	beta fibrinogen	C	0,9	2,0	1,6	1,8	1,6	0,8	0,9	1,3	2,5	2,3	1,5	2,0	3,5	1,0	2,6	0,4	0,4	0,6	0,7	0,3
93	WYSMZRMSMKIZPFF	beta fibrinogen	C	1,2	2,4	1,3	1,8	1,4	1,5	0,9	1,3	2,1	1,8	0,7	1,8	13,1	0,5	2,9	0,6	0,5	1,4	1,1	0,0
94	WYSMZZMSMKIRPFF	beta fibrinogen	C	1,2	2,1	1,3	2,1	1,6	1,0	0,9	1,1	1,6	2,4	0,8	2,5	12,7	1,6	2,9	0,6	0,6	1,4	1,2	0,0
95	WYSMZZMSMKIZPFF	beta fibrinogen	C	2,3	5,4	2,3	3,1	1,7	0,0	0,9	1,2	1,7	1,6	0,1	0,8	11,4	3,1	3,1	2,2	2,3	4,8	4,6	0,9
96	ZPAPPPISGGGYRAR	beta fibrinogen	C	0,6	1,9	1,5	5,9	3,0	1,0	0,7	1,0	2,5	1,2	1,2	1,5	8,5	2,3	10,9	0,4	0,6	3,9	3,3	0,2
97	ZPAPPPISGGGYRAZ	beta fibrinogen	C	1,3	2,5	3,1	4,7	3,8	1,0	0,7	1,2	3,0	1,6	1,1	1,8	8,8	2,0	4,1	0,7	1,5	5,1	3,6	0,7
98	ZPAPPPISGGGYZAR	beta fibrinogen	C	1,2	3,3	3,2	5,1	3,6	0,9	0,7	1,2	2,3	1,6	1,3	1,8	7,2	1,8	3,4	0,6	1,4	4,8	3,2	0,4
99	ZPAPPPISGGGYZAZ	beta fibrinogen	C	1,7	3,0	3,6	3,5	3,2	0,7	0,8	1,6	2,3	1,4	1,2	1,9	6,3	1,6	3,4	0,6	1,1	3,7	2,4	0,4
100	STRGRSRGRSGRSGS	filaggrin	R	1,8	2,4	3,9	4,5	2,5	0,9	0,9	1,4	2,2	2,1	2,0	2,9	10,4	1,5	12,5	2,2	1,4	9,9	9,9	0,4
101	STZGRSRGRSGRSGS	filaggrin	C	1,3	2,0	2,9	4,5	3,6	1,0	0,7	1,2	2,0	2,7	3,2	4,5	9,9	2,0	6,1	1,0	1,3	10,5	14,8	0,4
102	STZGZSRGRSGRSGS	filaggrin	C	1,3	2,5	2,8	3,7	3,2	1,0	0,7	1,2	2,7	1,3	1,3	1,9	5,3	1,8	7,5	0,4	0,6	4,3	4,9	0,2
103	STZGZSZGRSGRSGS	filaggrin	C	1,0	2,3	2,5	4,9	3,5	1,2	0,7	1,3	2,7	1,7	1,6	2,1	7,3	2,4	15,3	0,5	0,9	5,1	4,7	0,2
104	STZGZSZGZSGRSGS	filaggrin	C	1,3	2,5	3,1	4,3	2,9	0,9	0,7	1,2	1,3	1,5	1,2	1,6	7,6	2,2	7,5	0,5	1,0	4,7	3,5	0,3
105	STZGZSZGZSGZSGS	filaggrin	C	1,0	1,8	2,3	4,0	2,7	1,0	0,7	1,3	2,6	1,5	1,2	1,7	6,0	1,9	4,7	0,4	1,3	4,4	3,5	0,3
106	AIRRLARRGGVKRIS	histon 4	R	3,3	3,0	9,8	3,2	2,3	0,3	1,1	1,7	1,7	1,6	1,3	2,1	5,0	1,1	1,4	1,0	1,5	5,9	4,5	0,5
107	AIRRLARRGGVKZIS	histon 4	C	5,3	3,8	10,5	3,7	2,6	0,2	1,1	1,5	1,4	1,8	1,6	2,2	7,8	2,1	2,4	0,9	1,8	5,6	5,9	0,2
108	AIRRLARZGGVKRIS	histon 4	C	5,1	4,0	10,4	4,0	2,6	0,6	1,1	1,6	1,5	1,6	1,2	2,3	9,8	1,9	2,3	1,5	2,0	8,4	7,2	0,8
109	AIRRLAZRGGVKRIS	histon 4	C	5,2	3,7	9,6	3,9	2,7	0,5	1,2	1,8	1,3	1,4	1,3	2,3	11,6	2,2	2,6	1,2	2,0	8,7	7,1	0,9
110	AIRRLAZRGGVKZIS	histon 4	C	4,7	4,3	8,0	3,6	2,7	0,4	0,8	1,3	1,6	1,3	1,4	2,1	15,4	2,6	2,8	0,9	1,2	8,7	6,2	0,4
111	AIRRLAZZGGVKRIS	histon 4	C	3,3	2,6	6,1	2,9	2,6	0,6	1,3	1,5	2,3	1,3	1,1	2,2	25,6	3,8	4,8	1,1	2,0	11,3	7,3	0,6
112	AIRZLARRGGVKRIS	histon 4	C	4,1	3,2	8,1	3,1	2,5	0,5	1,1	1,5	1,5	1,5	1,3	2,3	10,5	2,3	2,2	1,1	1,5	7,6	6,9	0,5
113	AIRZLARRGGVKZIS	histon 4	C	6,7	4,5	12,1	3,5	2,5	0,2	1,0	1,4	1,4	1,4	1,3	2,0	11,7	3,0	2,4	0,9	1,0	7,0	5,5	0,0
114	AIRZLARZGGVKRIS	histon 4	C	8,9	5,6	14,7	3,4	2,5	0,1	1,3	1,7	1,7	1,4	1,3	2,2	15,0	2,9	3,9	1,1	1,4	8,0	6,4	0,1
115	AIRZLAZRGGVKRIS	histon 4	C	5,5	4,7	10,4	3,4	2,6	0,4	1,1	1,6	1,6	1,3	1,3	2,1	20,3	3,4	4,0	1,3	1,6	9,2	8,2	0,4
116	AIZRLARRGGVKRIS	histon 4	C	3,7	3,3	8,2	3,1	2,4	0,6	1,1	1,6	1,9	1,6	1,2	2,3	8,6	1,8	1,7	1,3	2,4	8,0	7,7	1,1
117	AIZRLARRGGVKZIS	histon 4	C	3,7	4,0	8,8	4,2	3,1	0,4	0,5	1,1	1,4	1,9	2,0	3,2	11,1	2,5	2,6	1,0	1,3	7,3	6,8	0,1
118	AIZRLARZGGVKRIS	histon 4	C	3,7	3,3	6,8	3,1	2,5	0,5	1,2	1,6	1,8	1,4	1,2	2,1	23,7	4,1	4,4	1,0	1,5	9,5	9,1	0,7
119	AIZRLAZRGGVKRIS	histon 4	C	3,9	3,4	6,9	3,2	2,6	0,5	1,0	1,5	1,8	1,4	1,3	2,2	16,6	3,1	3,5	1,1	1,6	8,3	8,5	0,5
120	AIZZLARRGGVKRIS	histon 4	C	6,5	4,5	11,6	2,7	2,2	0,3	1,3	1,8	2,0	1,5	1,4	2,0	11,0	2,7	2,8	1,2	1,8	9,1	8,3	0,7
121	AIZZLAZRGGVKRIS	histon 4	C	4,2	4,7	8,3	2,9	2,0	0,6	1,0	1,8	1,7	1,1	1,4	2,2	23,3	4,6	5,1	1,0	1,2	6,7	6,4	0,1
122	AIZZLAZZGGVKRIS	histon 4	C	2,0	5,2	7,8	3,3	2,1	0,6	0,8	1,6	1,7	1,4	2,7	2,3	11,3	2,7	5,2	0,7	0,8	2,9	2,5	0,2

123	AIZZLAZZGGVKZIS	histon 4	C	2,1	3,0	5,2	4,6	2,7	0,5	0,8	1,6	1,5	1,8	1,6	2,1	5,4	1,8	3,0	0,8	1,3	3,2	3,0	0,4
124	GAKRHRKVLRDNIQG	histon 4	R	1,6	2,8	3,4	1,7	1,4	0,9	0,9	1,4	2,3	2,2	1,7	2,3	4,9	1,3	2,3	0,2	0,6	0,8	0,9	0,2
125	GAKRHRKVLZDNIQG	histon 4	C	1,9	2,5	2,5	2,3	1,6	0,9	1,4	1,5	1,7	1,6	1,0	1,2	13,6	1,9	1,3	0,2	0,5	2,1	1,7	0,0
126	GAKRHZKVLRDNIQG	histon 4	C	1,6	2,9	2,5	1,7	1,3	0,7	1,4	1,3	1,8	1,7	1,1	1,7	18,0	4,0	2,8	0,2	0,6	2,4	2,6	0,1
127	GAKRHZKVLZDNIQG	histon 4	C	1,1	2,6	3,2	3,0	1,7	1,2	0,9	1,6	0,9	1,4	1,9	2,3	22,8	5,9	4,5	0,1	0,5	2,0	2,2	0,1
128	GAKZHRKVLRDNIQG	histon 4	C	1,9	2,7	2,9	1,7	1,3	1,2	1,4	1,8	1,7	1,4	1,1	1,5	16,3	4,1	2,6	0,2	0,6	2,3	2,5	0,2
129	GAKZHRKVLZDNIQG	histon 4	C	1,1	2,6	2,8	2,9	1,6	1,0	0,9	1,5	0,7	1,9	2,4	2,7	19,4	4,9	3,6	0,2	0,4	1,9	2,1	0,1
130	GAKZHZKVLRDNIQG	histon 4	C	0,8	2,0	2,5	1,7	1,0	1,3	0,8	1,2	1,3	1,3	1,6	1,4	26,7	5,0	4,7	0,1	0,3	2,3	2,4	0,1
131	GAKZHZKVLZDNIQG	histon 4	C	1,5	2,9	3,3	2,8	1,8	0,5	0,8	1,7	1,0	2,0	1,7	2,7	4,9	2,2	3,6	0,1	0,4	0,4	0,6	0,1
132	LRVTRGSRAPVSRAQ	proteoglycan	R	2,2	1,8	3,8	6,0	3,4	0,7	1,0	1,5	2,3	1,5	1,6	2,2	24,5	2,5	9,2	2,0	1,7	9,4	6,4	1,1
133	LRVTZGSRAPVSRAQ	proteoglycan	C	1,2	1,4	2,7	5,2	3,3	1,1	0,9	1,5	1,6	0,8	1,4	1,7	22,7	2,8	10,1	0,7	1,1	9,3	4,0	1,0
134	MDMCSAGWLADRSVR	proteoglycan	R	0,3	1,5	0,5	2,5	2,0	1,1	0,5	1,0	1,1	3,5	1,0	1,3	8,8	3,0	9,0	0,6	0,9	2,7	2,9	0,2
135	MDMCSAGWLADRSVZ	proteoglycan	C	0,6	2,5	1,2	4,2	2,6	0,7	0,6	1,0	2,1	2,4	1,2	1,8	4,0	1,8	6,3	0,9	1,4	1,9	4,0	0,3
136	MDMCSAGWLADZSVR	proteoglycan	C	0,5	2,1	1,2	2,8	2,2	1,3	0,6	1,0	1,1	3,2	1,8	2,3	4,9	3,4	6,1	0,8	1,5	2,5	4,7	0,1
137	MDMCSAGWLADZSVZ	proteoglycan	C	0,8	2,7	1,4	4,0	2,3	0,9	0,5	1,1	2,1	1,8	1,4	2,3	3,9	1,4	5,6	1,0	1,7	1,9	3,7	0,3
138	AYVTRSSAVRLRSSV	vimentin	R	5,1	3,8	6,9	2,7	3,5	1,9	2,3	0,2	2,5	1,6	1,4	2,4	4,6	1,0	1,1	1,7	2,8	4,6	4,7	1,1
139	AYVTRSSAVRLZSSV	vimentin	C	3,1	4,8	4,9	2,2	3,2	1,3	1,1	1,3	2,5	1,3	1,5	2,4	3,5	0,9	1,2	1,4	2,7	5,3	5,3	1,3
140	AYVTRSSAVZLRSSV	vimentin	C	2,1	3,6	3,8	1,9	3,6	1,7	1,0	1,2	2,5	1,4	1,6	2,3	7,8	2,6	4,5	1,6	1,8	4,8	3,7	0,9
141	AYVTRSSAVZLZSSV	vimentin	C	0,9	5,4	1,8	2,6	4,5	2,2	1,0	1,4	2,1	1,1	1,0	1,0	3,2	2,6	6,7	1,0	1,3	2,3	4,3	0,8
142	AYVTRSSAVZLZSSVP	vimentin	C	1,2	7,2	2,7	2,2	4,0	1,7	0,5	1,7	2,7	1,8	1,2	2,2	3,3	2,5	4,3	1,0	2,2	1,7	2,7	0,9
143	AYVTZSSAVRLRSSV	vimentin	C	3,5	4,8	4,8	2,0	2,9	1,1	1,0	1,3	2,4	1,3	1,3	2,2	10,9	2,1	3,6	1,1	2,2	4,3	1,5	0,7
144	AYVTZSSAVRLZSSV	vimentin	C	1,2	6,2	2,2	3,0	4,6	1,4	0,7	0,6	2,7	1,4	1,3	1,5	3,4	2,8	7,1	1,2	2,5	2,2	7,0	1,1
145	AYVTZSSAVZLRSSV	vimentin	C	1,7	6,7	3,0	2,8	4,3	1,6	1,3	0,9	2,5	1,8	1,7	1,9	3,9	2,2	3,4	0,8	1,1	2,0	3,4	0,9
146	AYVTZSSAVZLZSSV	vimentin	C	1,3	6,9	2,7	3,0	4,5	1,8	0,7	1,3	2,6	2,4	1,2	1,6	3,4	2,5	3,9	0,9	1,5	2,2	4,5	1,0
147	AYVTZSSAVZLZSSVP	vimentin	C	1,0	7,4	2,1	2,4	3,9	1,3	0,7	1,7	2,8	2,0	1,4	2,0	2,8	1,3	8,3	1,2	2,1	1,3	7,2	0,7
148	SAVRLRSSVPGVRLL	vimentin	R	3,5	3,5	6,1	3,0	2,4	1,8	0,9	1,8	1,9	1,7	1,3	2,4	10,7	0,1	0,1	0,0	0,2	0,8	0,5	0,0
149	SAVRLRSSVPGVZLL	vimentin	C	2,8	3,4	4,1	3,2	2,3	2,0	1,3	2,1	2,8	1,4	1,3	2,3	9,3	1,3	1,0	0,5	0,6	2,1	2,1	0,2
150	SAVRLZSSVPGVRLL	vimentin	C	2,0	3,1	3,7	2,8	2,2	1,6	0,6	1,8	1,7	1,5	1,4	2,2	10,6	1,6	1,7	0,5	0,8	2,9	2,8	0,2
151	SAVRLZSSVPGVZLL	vimentin	C	1,1	2,9	2,6	2,7	2,3	0,8	0,6	1,4	1,9	3,6	2,4	3,9	0,7	1,1	0,9	0,0	0,5	0,2	1,2	0,2
152	SAVZLRSSVPGVRLL	vimentin	C	2,1	2,9	3,8	2,8	2,2	2,0	0,9	2,1	1,6	1,5	1,3	2,1	10,6	1,8	1,7	1,6	1,8	6,9	6,5	0,5
153	SAVZLRSSVPGVZLL	vimentin	C	0,9	3,4	2,1	2,8	2,5	2,2	0,6	1,4	1,8	2,0	1,9	1,8	1,2	0,9	1,8	0,7	1,2	1,1	1,7	0,4
154	SAVZLZSSVPGVRLL	vimentin	C	1,4	3,6	3,3	3,9	2,5	1,0	0,7	1,6	1,7	2,1	1,4	2,2	0,7	0,4	0,7	0,6	0,3	0,6	0,7	0,3
155	SAVZLZSSVPGVZLL	vimentin	C	1,1	2,6	2,0	3,5	2,5	1,4	0,7	1,3	1,6	2,6	1,4	0,7	0,9	1,0	1,0	1,2	2,1	1,3	3,3	1,1
156	STRSVSSSSYRRMFG	vimentin	R	3,8	3,2	5,0	3,3	3,4	0,5	1,0	2,3	2,0	1,3	0,9	2,1	12,8	1,9	2,2	1,3	1,9	5,7	5,1	0,5
157	STRSVSSSSYRZMFG	vimentin	C	4,1	4,3	4,7	1,7	3,5	0,6	0,9	2,2	2,4	1,2	1,0	2,0	14,5	3,7	3,8	1,8	2,6	4,8	4,7	0,4
158	STRSVSSSSYZRMFG	vimentin	C	4,6	4,3	6,1	2,2	3,9	0,4	0,8	2,3	2,1	1,3	1,0	2,1	24,0	3,0	3,5	2,6	2,2	7,4	4,6	0,1
159	STRSVSSSSYZZMFG	vimentin	C	1,9	3,5	3,1	2,2	4,7	1,1	0,9	1,4	2,0	1,0	1,0	1,9	3,8	1,4	2,9	1,3	2,1	1,6	2,1	0,8
160	STZSVSSSSYRRMFG	vimentin	C	3,9	3,8	4,8	2,3	4,4	0,7	1,1	2,0	2,4	1,3	0,9	2,1	14,6	3,0	2,6	2,2	2,2	4,9	4,9	0,5
161	STZSVSSSSYRZMFG	vimentin	C	1,3	3,1	1,9	2,0	4,8	1,1	0,8	1,5	2,2	0,9	1,1	1,4	8,0	1,9	4,2	1,4	2,6	3,7	3,2	0,5
162	STZSVSSSSYZRMFG	vimentin	C	1,4	2,6	2,1	2,4	5,6	1,1	1,1	1,5	2,3	1,0	1,1	1,6	9,4	1,5	2,9	3,1	2,5	4,2	3,0	0,4
163	STZSVSSSSYZZMFG	vimentin	C	1,9	4,3	2,8	2,2	4,8	1,1	0,7	1,1	2,2	1,6	1,0	1,8	2,8	2,0	3,5	0,8	2,6	1,4	3,3	0,3
164	YVTRSSAVRLRSSVP	vimentin	R	3,4	2,9	4,5	2,4	3,7	1,1	0,9	1,8	2,3	1,7	1,4	2,5	6,1	1,6	1,9	1,6	2,7	6,3	6,8	1,0
165	YVTRSSAVRLZSSVP	vimentin	C	2,3	2,9	3,1	1,9	3,5	1,4	0,9	1,6	2,4	1,4	1,7	2,4	6,1	1,0	2,3	1,3	2,2	4,9	4,2	0,6
166	YVTRSSAVZLRSSVP	vimentin	C	1,8	2,3	2,8	2,3	3,4	1,4	0,8	1,6	2,8	1,5	1,8	2,4	32,0	4,4	17,7	1,4	1,6	3,1	3,7	0,3
167	YVTZSSAVRLRSSVP	vimentin	C	2,6	2,8	3,1	2,1	3,1	1,2	0,9	1,4	3,1	1,6	1,7	2,6	0,0	0,0	0,0	0,6	1,4	3,5	4,7	0,0
168	YVTZSSAVRLZSSVP	vimentin	C	1,2	3,5	2,1	2,3	3,9	1,4	0,8	1,1	3,2	1,6	1,2	2,2	2,2	1,7	2,8	0,9	2,5	1,3	4,3	1,0
169	YVTZSSAVZLRSSVP	vimentin	C	1,0	2,9	1,9	2,5	4,3	1,3	0,9	1,1	2,7	1,3	1,1	2,0	3,2	3,5	6,9	1,1	1,5	1,0	3,2	0,8

Positive sera to arginine peptide
Positive sera to citrullinated peptide
Positive sera specific to citrullinated peptide
