# Supplementary material for: PAD4 Immunization Triggers Anti-Citrullinated Peptide Antibodies in Normal Mice: Analysis With Peptide Arrays
Source: Front Immunol. 2022 Mar 31;13:840035. doi: 10.3389/fimmu.2022.840035 (PMC9008206; doi:10.3389/fimmu.2022.840035)
Supplement: Supplementary file 2 [file Image_2.pdf]

Supplementary figure 2: Ratios for mice immunized with PBS

PBS immunization

Peptide number	Sequence	Protein	Arginine (R) citrulline (C)	C3H-6	C3H-7	C3H-8	C3H-9	C3H-10	DBA/2-5	DBA/2-6	DBA/2-7	DBA/2-8	DBA/2-9	BALB/c-4	BALB/c-5	BALB/c-6	BALB/c-7	BALB/c-8	BL6-9	BL6-10	BL6-11	BL6-12	BL6-13	BL6-14
1	GARGLTGNPGVQGPE	collagen	R	1,3	0,7	1,0	0,8	1,2	0,9	1,1	2,3	0,4	0,3	1,3	0,7	0,9	0,6	0,0	0,2	1,8	1,8	0,0	0,0	2,2
2	GAZGLTGNPGVQGPE	collagen	C	1,3	0,7	1,5	1,1	0,4	0,9	1,1	2,1	0,5	0,4	1,4	0,6	0,4	1,0	0,1	0,2	1,8	2,4	0,1	0,2	1,2
3	SRGQSRGRGRGRGRG	ebna 2	R	1,1	0,9	2,0	1,0	0,0	0,9	1,1	2,2	0,6	0,2	1,5	0,5	0,3	0,8	0,2	0,5	1,5	3,8	0,0	0,0	0,3
4	SZGQSZGZGZGZGZG	ebna 2	C	1,4	0,6	1,9	1,0	0,2	0,9	1,1	2,1	0,6	0,3	1,5	0,5	0,1	0,4	0,0	0,2	1,8	4,0	0,0	0,0	0,1
5	IHAREIFDSRGNPTV	enolase	R	0,7	1,3	1,7	0,7	0,6	1,0	1,0	1,3	1,1	0,6	1,4	0,6	1,1	0,5	1,2	0,2	1,8	0,9	0,4	1,3	1,5
6	IHAREIFDSZGNPTV	enolase	C	0,8	1,2	1,7	0,9	0,4	0,9	1,1	2,1	0,4	0,5	1,3	0,7	1,1	0,5	0,9	0,1	1,9	1,7	0,0	0,8	1,6
7	IHAZEIFDSRGNPTV	enolase	C	0,8	1,2	1,9	0,8	0,3	0,9	1,1	1,9	0,7	0,5	1,3	0,7	1,2	0,3	1,2	0,1	1,9	1,1	0,3	1,3	1,3
8	IHAZEIFDSZGNPTV	enolase	C	0,9	1,1	1,7	1,0	0,4	0,9	1,1	2,5	0,3	0,2	1,4	0,6	0,5	0,6	1,0	0,1	1,9	2,1	0,1	0,7	1,2
9	RIHAREIFDSRGNPT	enolase	R	1,0	1,0	1,5	0,7	0,8	1,1	0,9	1,5	0,9	0,6	1,4	0,6	0,9	1,0	0,9	0,3	1,7	1,8	0,0	1,0	1,2
10	RIHAREIFDSZGNPT	enolase	C	0,8	1,2	1,5	1,1	0,4	0,9	1,1	1,8	0,6	0,5	1,4	0,6	1,2	0,7	0,8	0,1	1,9	0,9	0,3	1,5	1,3
11	RIHAZEIFDSRGNPT	enolase	C	0,7	1,3	1,6	0,8	0,6	0,9	1,1	1,7	0,8	0,6	1,3	0,7	1,1	0,5	1,2	0,1	1,9	0,6	0,3	1,6	1,5
12	RIHAZEIFDSZGNPT	enolase	C	1,0	1,0	1,8	1,0	0,2	1,0	1,0	2,0	0,4	0,6	1,4	0,6	1,1	0,5	1,1	0,3	1,7	1,0	0,2	1,2	1,7
13	TAKGLFRAAVPSGAS	enolase	R	1,1	0,9	0,9	1,0	1,1	0,9	1,1	1,9	0,4	0,7	1,5	0,5	0,9	0,5	0,9	0,1	1,9	1,0	0,3	0,9	1,8
14	TAKGLFZAAVPSGAS	enolase	C	1,1	0,9	1,2	0,8	1,0	0,8	1,2	2,0	0,6	0,4	1,3	0,7	0,3	0,4	0,9	0,1	1,9	1,7	0,2	0,8	1,3
15	ZIHAREIFDSRGNPT	enolase	C	1,0	1,0	1,6	0,8	0,5	0,9	1,1	2,1	0,2	0,7	1,4	0,6	0,4	1,8	0,3	0,2	1,8	1,9	0,1	0,5	1,5
16	ZIHAREIFDSZGNPT	enolase	C	1,0	1,0	1,8	1,1	0,2	1,0	1,0	2,4	0,3	0,3	1,4	0,6	0,4	1,2	0,6	0,1	1,9	2,0	0,0	0,4	1,6
17	ZIHAZEIFDSRGNPT	enolase	C	1,0	1,0	1,6	1,0	0,4	0,9	1,1	2,5	0,2	0,3	1,4	0,6	0,4	1,0	0,7	0,1	1,9	1,9	0,0	0,7	1,4
18	ZIHAZEIFDSZGNPT	enolase	C	1,1	0,9	1,9	1,0	0,1	0,9	1,1	2,2	0,4	0,4	1,5	0,5	0,6	0,7	0,6	0,2	1,8	2,3	0,0	0,5	1,1
19	GGGVRGPRVVERHQS	alpha fibrinogen	R	1,0	1,0	2,1	0,6	0,3	1,0	1,0	1,6	0,7	0,7	1,6	0,4	0,3	1,3	1,1	0,1	1,9	1,8	0,0	0,8	1,3
20	GGGVRGPRVVEZHQS	alpha fibrinogen	C	0,7	1,3	1,8	0,8	0,4	0,9	1,1	1,8	0,4	0,8	1,6	0,4	0,1	2,1	0,1	0,1	1,9	2,8	0,0	0,0	1,1
21	GGGVRGPZVVERHQS	alpha fibrinogen	C	0,6	1,4	1,9	0,7	0,4	0,8	1,2	1,9	0,7	0,4	1,6	0,4	0,2	1,5	0,3	0,1	1,9	2,5	0,0	0,1	1,4
22	GGGVRGPZVVEZHQS	alpha fibrinogen	C	0,9	1,1	1,8	1,1	0,1	0,8	1,2	2,4	0,3	0,3	1,4	0,6	0,2	0,9	0,0	0,1	1,9	3,8	0,0	0,0	0,2
23	GGGVZGPRVVERHQS	alpha fibrinogen	C	0,8	1,2	2,1	0,7	0,2	0,9	1,1	2,3	0,3	0,4	1,6	0,4	0,4	0,7	0,5	0,1	1,9	1,5	0,0	0,5	2,0
24	GGGVZGPRVVEZHQS	alpha fibrinogen	C	0,9	1,1	1,9	0,9	0,2	0,9	1,1	2,1	0,5	0,4	1,5	0,5	0,3	0,6	0,9	0,1	1,9	3,6	0,0	0,0	0,4
25	GGGVZGPZVVERHQS	alpha fibrinogen	C	1,1	0,9	2,1	0,8	0,2	0,8	1,2	2,0	0,7	0,3	1,5	0,5	0,4	0,6	0,4	0,3	1,7	2,8	0,0	0,0	1,2
26	GGGVZGPZVVEZHQS	alpha fibrinogen	C	0,9	1,1	1,9	1,0	0,1	0,7	1,3	2,3	0,4	0,3	1,5	0,5	0,3	0,4	0,3	0,1	1,9	3,8	0,0	0,0	0,1
27	GPRVVERHQSQCKDS	alpha fibrinogen	R	1,0	1,0	1,7	0,8	0,5	0,8	1,2	1,6	0,9	0,5	1,4	0,6	0,9	0,3	1,0	0,1	1,9	0,3	0,2	1,3	2,2
28	GPRVVEZHQSQCKDS	alpha fibrinogen	C	1,2	0,8	1,6	0,8	0,6	1,1	0,9	2,1	0,4	0,5	1,5	0,5	0,7	0,6	1,1	0,1	1,9	0,6	0,1	1,0	2,3
29	GPZVVERHQSQCKDS	alpha fibrinogen	C	0,9	1,1	2,0	0,8	0,2	0,9	1,1	2,1	0,4	0,5	1,3	0,7	0,4	0,7	0,4	0,1	1,9	1,1	0,0	0,7	2,2
30	GPZVVEZHQSQCKDS	alpha fibrinogen	C	1,1	0,9	2,1	0,8	0,2	0,9	1,1	1,9	0,7	0,4	1,5	0,5	0,4	0,8	0,6	0,3	1,7	1,7	0,1	0,6	1,6
31	MELERPGKDGGSRGD	alpha fibrinogen	R	0,9	1,1	1,7	1,1	0,2	1,0	1,0	2,1	0,4	0,4	1,5	0,5	0,1	0,7	0,2	0,3	1,7	2,3	0,0	0,0	1,7
32	MELERPGKDGGSZGD	alpha fibrinogen	C	0,9	1,1	1,9	1,0	0,1	0,9	1,1	2,2	0,4	0,4	1,5	0,5	0,4	0,6	0,5	0,1	1,9	2,0	0,0	0,1	1,8
33	MELEZPGKDGGSRGD	alpha fibrinogen	C	1,1	0,9	1,7	1,0	0,3	1,0	1,0	2,3	0,4	0,3	1,5	0,5	0,2	0,6	0,4	0,1	1,9	2,4	0,0	0,1	1,5
34	MELEZPGKDGGSZGD	alpha fibrinogen	C	1,0	1,0	1,7	1,2	0,1	0,8	1,2	2,2	0,5	0,3	1,4	0,6	0,2	0,8	0,4	0,3	1,7	2,2	0,1	0,2	1,5
35	SCSRAVNREINLQDY	alpha fibrinogen	R	1,0	1,0	1,7	0,9	0,4	0,9	1,1	1,9	0,6	0,5	0,8	1,2	0,9	1,0	0,7	0,2	1,8	1,5	0,2	0,8	1,5
36	SCSRAVNZEINLQDY	alpha fibrinogen	C	1,1	0,9	1,7	1,0	0,2	0,8	1,2	1,9	0,5	0,6	1,4	0,6	0,4	0,9	0,3	0,0	2,0	3,7	0,1	0,1	0,1
37	SCSZAVNREINLQDY	alpha fibrinogen	C	0,9	1,1	2,1	0,9	0,0	0,9	1,1	2,5	0,3	0,2	1,5	0,5	0,3	0,6	0,5	0,0	2,0	2,4	0,0	0,5	1,2
38	SCSZAVNZEINLQDY	alpha fibrinogen	C	0,9	1,1	1,7	1,1	0,2	0,8	1,2	2,2	0,5	0,3	1,5	0,5	0,1	0,7	0,1	0,1	1,9	3,9	0,0	0,0	0,1
39	APPPISGGGYRARPA	beta fibrinogen	R	1,3	0,7	1,3	1,1	0,6	0,9	1,1	2,3	0,4	0,3	1,5	0,5	0,7	0,7	0,3	0,1	1,9	3,4	0,0	0,1	0,5
40	APPPISGGGYRAZPA	beta fibrinogen	C	1,3	0,7	1,5	1,3	0,3	0,9	1,1	2,0	0,7	0,3	1,5	0,5	0,1	0,8	0,4	0,1	1,9	3,9	0,0	0,0	0,1
41	APPPISGGGYZARPA	beta fibrinogen	C	1,4	0,6	1,4	1,2	0,4	1,0	1,0	2,2	0,5	0,4	1,4	0,6	0,3	0,6	0,2	0,0	2,0	3,6	0,0	0,1	0,3
42	APPPISGGGYZAZPA	beta fibrinogen	C	1,3	0,7	1,8	1,0	0,2	0,9	1,1	2,2	0,5	0,2	1,4	0,6	0,1	0,8	0,3	0,2	1,8	3,7	0,0	0,1	0,2
43	FSTYDRDNDGWVTTD	beta fibrinogen	R	1,6	0,4	1,7	1,3	0,1	0,7	1,3	2,5	0,3	0,2	0,8	1,2	0,3	0,7	0,1	0,3	1,7	2,2	0,0	0,4	1,4
44	FSTYDZDNDGWVTTD	beta fibrinogen	C	1,6	0,4	1,7	1,2	0,1	0,9	1,1	2,4	0,3	0,3	0,9	1,1	0,6	0,5	0,3	0,2	1,8	2,7	0,0	0,1	1,1
45	GSWYSMRRMSMKIRP	beta fibrinogen	R	0,9	1,1	1,4	1,6	0,0	0,8	1,2	2,2	0,5	0,3	1,5	0,5	1,1	0,0	1,0	0,0	2,4	1,3	0,8	1,2	0,7
46	GSWYSMRRMSMKIZP	beta fibrinogen	C	0,7	1,3	1,8	1,0	0,2	0,7	1,3	1,8	0,7	0,5	1,0	1,0	0,8	0,4	1,1	0,0	2,0	0,6	0,2	1,5	1,6
47	GSWYSMRZMSMKIRP	beta fibrinogen	C	0,8	1,2	1,8	1,2	0,1	0,7	1,3	2,3	0,4	0,2	1,1	0,9	0,8	0,3	0,6	0,3	1,7	1,3	0,2	1,7	0,8
48	GSWYSMZRMSMKIRP	beta fibrinogen	C	0,9	1,1	1,9	1,1	0,0	1,0	1,0	2,2	0,4	0,4	1,4	0,6	0,9	0,0	0,8	0,1	1,9	1,3	0,2	1,4	1,2
49	GSWYSMZZMSMKIZP	beta fibrinogen	C	0,9	1,1	1,7	0,8	0,4	1,0	1,0	2,0	0,5	0,5	1,4	0,6	1,2	0,3	1,0	0,2	1,8	1,0	0,2	1,3	1,6
50	LVGENRTMTIHNGMF	beta fibrinogen	R	1,4	0,6	1,2	0,9	0,9	1,0	1,0	1,1	1,5	0,4	1,3	0,7	1,3	0,6	0,9	0,3	1,7	1,1	0,2	1,0	1,7
51	LVGENZTMTIHNGMF	beta fibrinogen	C	1,5	0,5	1,4	1,0	0,6	0,9	1,1	1,5	1,1	0,4	1,3	0,7	1,1	0,7	1,0	0,2	1,8	1,4	0,2	0,9	1,5
52	MRRMSMKIRPFFPQQ	beta fibrinogen	R	1,4	0,6	1,8	1,0	0,2	0,9	1,1	1,5	0,8	0,7	1,0	1,0	0,8	0,9	0,8	0,4	1,6	1,7	0,2	0,8	1,3
53	MRRMSMKIZPFFPQQ	beta fibrinogen	C	1,7	0,3	1,7	1,1	0,3	0,9	1,1	1,7	0,7	0,5	1,0	1,0	0,8	0,8	1,0	0,3	1,7	2,2	0,0	0,7	1,1
54	MRZMSMKIRPFFPQQ	beta fibrinogen	C	1,7	0,3	1,7	1,0	0,3	0,8	1,2	1,9	0,6	0,5	1,0	1,0	0,7	1,0	1,0	0,3	1,7	1,9	0,1	0,6	1,4
55	MRZMSMKIZPFFPQQ	beta fibrinogen	C	1,7	0,3	1,6	1,2	0,1	0,7	1,3	2,2	0,5	0,3	1,0	1,0	0,1	1,3	1,1	0,1	1,9	2,8	0,0	0,2	0,9
56	MZRMSMKIRPFFPQQ	beta fibrinogen	C	1,4	0,6	1,8	0,9	0,2	0,9	1,1	1,5	0,9	0,6	1,0	1,0	0,8	0,8	0,9	0,3	1,7	1,9	0,0	0,6	1,4
57	MZRMSMKIZPFFPQQ	beta fibrinogen	C	1,9	0,1	1,5	1,0	0,5	0,9	1,1	2,1	0,6	0,3	0,9	1,1	0,6	1,0	0,9	0,2	1,8	2,8	0,0	0,3	0,9

58	MZZMSMKIRPFFPQQ	beta fibrinogen	C	1,8	0,2	1,4	1,2	0,4	1,0	1,0	1,7	0,8	0,5	1,1	0,9	0,8	0,9	1,0	0,3	1,7	1,7	0,0	0,8	1,5
59	MZZMSMKIZPFFPQQ	beta fibrinogen	C	1,7	0,3	1,8	1,1	0,1	0,7	1,3	2,2	0,5	0,3	0,8	1,2	0,5	0,9	0,3	0,2	1,8	3,3	0,0	0,0	0,7
60	NRCHAANPNGRYYWG	beta fibrinogen	R	1,2	0,8	1,9	1,1	0,1	0,8	1,2	2,5	0,4	0,2	0,8	1,2	1,1	0,7	0,7	0,0	3,3	3,3	0,1	0,3	0,3
61	NRCHAANPNGZYYWG	beta fibrinogen	C	1,3	0,7	1,8	1,1	0,1	0,9	1,1	2,2	0,5	0,3	0,8	1,2	0,8	0,7	1,1	0,0	2,0	3,7	0,0	0,1	0,1
62	NZCHAANPNGRYYWG	beta fibrinogen	C	1,5	0,5	1,9	1,0	0,1	1,0	1,0	2,3	0,5	0,3	0,8	1,2	0,4	1,3	0,3	0,0	2,0	3,6	0,0	0,1	0,3
63	NZCHAANPNGZYYWG	beta fibrinogen	C	1,4	0,6	1,9	1,0	0,1	1,0	1,0	2,1	0,6	0,3	0,7	1,3	0,3	1,1	0,8	0,0	2,0	3,5	0,0	0,0	0,5
64	PRKQCSKEDGGGWWY	beta fibrinogen	R	1,6	0,4	1,8	1,0	0,2	1,0	1,0	0,9	2,0	0,1	0,7	1,3	0,5	0,6	0,8	0,3	1,7	3,7	0,0	0,1	0,2
65	PZKQCSKEDGGGWWY	beta fibrinogen	C	1,8	0,2	1,7	1,0	0,3	1,0	1,0	0,8	2,2	0,1	0,9	1,1	0,3	0,6	0,4	0,3	1,7	3,6	0,0	0,2	0,3
66	QKLESDISAQMEYCR	beta fibrinogen	R	1,6	0,4	2,0	0,8	0,2	1,0	1,0	2,5	0,3	0,2	1,3	0,7	0,4	0,5	1,0	0,3	1,7	2,2	0,0	0,3	1,4
67	QKLESDISAQMEYCZ	beta fibrinogen	C	1,6	0,4	1,9	0,9	0,2	0,9	1,1	2,3	0,3	0,4	1,3	0,7	0,3	1,0	0,3	0,2	1,8	3,6	0,1	0,1	0,2
68	RPAPPPISGGGYRAR	beta fibrinogen	R	0,9	1,1	1,6	1,3	0,2	0,8	1,2	1,4	1,3	0,3	1,5	0,5	0,2	0,6	0,3	0,0	2,0	3,8	0,0	0,0	0,2
69	RPAPPPISGGGYRAZ	beta fibrinogen	C	0,9	1,1	1,8	1,0	0,3	1,0	1,0	2,1	0,6	0,3	1,5	0,5	0,4	0,7	0,4	0,2	1,8	3,7	0,1	0,1	0,2
70	RPAPPPISGGGYZAR	beta fibrinogen	C	1,0	1,0	1,9	0,7	0,3	0,9	1,1	1,7	0,8	0,5	1,5	0,5	0,2	0,9	0,5	0,1	1,9	3,8	0,0	0,0	0,2
71	RPAPPPISGGGYZAZ	beta fibrinogen	C	1,6	0,4	1,4	1,2	0,4	0,9	1,1	1,9	0,7	0,4	1,4	0,6	0,4	0,9	0,4	0,2	1,8	3,7	0,1	0,0	0,1
72	VIQNRQDGSVDFGRK	beta fibrinogen	R	1,2	0,8	1,1	1,1	0,7	1,0	1,0	2,4	0,4	0,2	1,6	0,4	0,5	0,9	0,5	0,1	1,9	3,2	0,0	0,6	0,2
73	VIQNRQDGSVDFGZK	beta fibrinogen	C	1,3	0,7	1,8	0,9	0,3	0,9	1,1	2,2	0,5	0,2	1,5	0,5	0,4	0,9	0,4	0,2	1,8	3,6	0,1	0,1	0,3
74	VIQNZQDGSVDFGRK	beta fibrinogen	C	1,4	0,6	1,6	1,1	0,3	0,9	1,1	2,2	0,4	0,4	1,5	0,5	0,3	0,9	0,3	0,3	1,7	3,6	0,0	0,2	0,2
75	VIQNZQDGSVDFGZK	beta fibrinogen	C	1,3	0,7	1,8	1,0	0,2	0,9	1,1	2,2	0,5	0,2	1,4	0,6	0,3	0,5	0,6	0,2	1,8	3,9	0,0	0,0	0,1
76	VTTDPRKQCSKEDGG	beta fibrinogen	R	1,2	0,8	1,7	1,0	0,3	0,9	1,1	2,1	0,5	0,3	1,4	0,6	0,2	0,7	0,4	0,3	1,7	2,5	0,0	0,2	1,3
77	VTTDPZKQCSKEDGG	beta fibrinogen	C	1,2	0,8	1,7	1,0	0,3	1,0	1,0	1,9	0,6	0,5	1,4	0,6	0,3	0,5	0,4	0,3	1,7	3,1	0,0	0,2	0,7
78	VVWMNWKGSWYSMRK	beta fibrinogen	R	1,0	1,0	1,9	0,9	0,2	0,9	1,1	2,1	0,7	0,3	1,3	0,7	0,6	0,5	0,9	0,0	2,0	1,4	0,0	0,9	1,7
79	VVWMNWKGSWYSMRF	beta fibrinogen	R	0,9	1,1	1,9	0,9	0,2	0,8	1,2	1,5	1,3	0,3	1,4	0,6	0,8	0,7	1,1	0,0	2,2	2,1	0,1	0,7	1,0
80	VVWMNWKGSWYSMRZ	beta fibrinogen	C	1,3	0,7	2,0	0,8	0,3	0,9	1,1	1,4	1,4	0,2	0,9	1,1	0,9	0,7	0,9	0,0	2,5	2,4	0,1	0,8	0,7
81	VVWMNWKGSWYSMZK	beta fibrinogen	C	1,6	0,4	1,5	1,2	0,3	1,0	1,0	1,4	1,5	0,1	1,1	0,9	1,0	0,5	0,9	0,1	1,9	1,7	0,1	0,7	1,6
82	VVWMNWKGSWYSMZR	beta fibrinogen	C	1,4	0,6	1,9	0,9	0,3	0,8	1,2	1,5	1,3	0,2	1,0	1,0	1,1	0,4	0,8	0,0	4,8	1,6	0,1	0,9	1,4
83	VVWMNWKGSWYSMZZ	beta fibrinogen	C	1,2	0,8	1,8	0,9	0,3	0,9	1,1	1,4	1,4	0,1	0,9	1,1	1,0	0,7	0,9	0,0	2,0	2,3	0,0	0,5	1,2
84	WYNRCHAANPNGRYY	beta fibrinogen	R	1,8	0,2	1,4	1,3	0,3	1,2	0,8	2,0	0,7	0,3	1,2	0,8	0,7	1,0	1,2	2,1	0,0	2,2	0,1	0,7	1,0
85	WYNRCHAANPNGZYY	beta fibrinogen	C	1,3	0,7	1,6	1,2	0,2	1,0	1,0	2,0	0,7	0,3	0,8	1,2	0,9	0,9	1,0	0,2	1,8	2,8	0,0	0,3	0,8
86	WYNZCHAANPNGRYY	beta fibrinogen	C	1,3	0,7	1,8	1,1	0,1	0,9	1,1	2,1	0,6	0,2	0,9	1,1	0,9	0,9	1,0	0,3	1,7	3,1	0,0	0,3	0,6
87	WYNZCHAANPNGZYY	beta fibrinogen	C	1,2	0,8	1,7	1,2	0,1	1,0	1,0	2,3	0,5	0,2	0,9	1,1	1,3	0,5	0,8	0,0	2,0	1,2	0,0	0,5	2,2
88	WYSMRRMSMKIRPFF	beta fibrinogen	R	0,8	1,2	1,8	0,9	0,4	1,0	1,0	1,7	0,8	0,6	1,5	0,5	0,7	0,2	1,5	0,3	1,7	1,8	0,2	1,0	1,1
89	WYSMRRMSMKIZPFF	beta fibrinogen	C	1,1	0,9	1,8	1,0	0,2	1,1	0,9	2,0	0,6	0,4	1,1	0,9	0,8	0,8	0,8	0,2	1,8	1,2	0,2	1,8	0,8
90	WYSMRZMSMKIRPFF	beta fibrinogen	C	0,8	1,2	2,0	0,9	0,1	0,8	1,2	2,3	0,4	0,3	1,3	0,7	0,7	0,5	0,9	0,2	1,8	2,3	0,2	0,9	0,6
91	WYSMRZMSMKIZPFF	beta fibrinogen	C	1,8	0,2	1,6	0,8	0,5	0,7	1,3	1,9	0,7	0,4	1,1	0,9	0,9	0,7	1,2	0,0	2,7	1,2	0,1	1,0	1,7
92	WYSMZRMSMKIRPFF	beta fibrinogen	C	0,9	1,1	1,9	0,8	0,3	1,0	1,0	2,0	0,6	0,4	1,4	0,6	0,9	0,3	1,3	0,0	1,8	1,2	0,2	1,5	1,1
93	WYSMZRMSMKIZPFF	beta fibrinogen	C	1,2	0,8	1,9	0,9	0,3	1,1	0,9	2,1	0,5	0,4	1,2	0,8	0,9	0,7	1,1	0,0	2,6	1,4	0,1	1,1	1,4
94	WYSMZZMSMKIRPFF	beta fibrinogen	C	1,4	0,6	1,8	1,0	0,3	0,8	1,2	2,0	0,6	0,4	1,2	0,8	0,7	0,7	1,4	0,0	2,5	1,8	0,1	0,8	1,3
95	WYSMZZMSMKIZPFF	beta fibrinogen	C	1,9	0,1	1,4	1,1	0,5	0,8	1,2	2,1	0,5	0,4	0,8	1,2	1,0	0,8	0,9	0,2	1,8	2,7	0,1	0,4	0,8
96	ZPAPPPISGGGYRAR	beta fibrinogen	C	1,0	1,0	2,0	0,8	0,3	0,8	1,2	1,9	0,8	0,4	1,6	0,4	0,3	0,7	0,4	0,1	1,9	3,7	0,0	0,0	0,2
97	ZPAPPPISGGGYRAZ	beta fibrinogen	C	1,3	0,7	1,8	1,1	0,1	0,9	1,1	2,0	0,7	0,3	1,5	0,5	0,1	0,8	0,0	0,3	1,7	3,8	0,0	0,0	0,2
98	ZPAPPPISGGGYZAR	beta fibrinogen	C	1,3	0,7	1,9	0,8	0,2	1,0	1,0	2,2	0,6	0,2	1,5	0,5	0,4	0,5	0,1	0,2	1,8	3,8	0,0	0,0	0,2
99	ZPAPPPISGGGYZAZ	beta fibrinogen	C	1,4	0,6	1,6	1,3	0,1	0,9	1,1	2,3	0,5	0,2	1,5	0,5	0,0	0,4	0,0	0,3	1,7	4,0	0,0	0,0	0,1
100	STRGRSRGRSGRSGS	filaggrin	R	0,8	1,2	2,1	0,8	0,1	1,0	1,0	2,2	0,4	0,3	1,4	0,6	0,0	0,7	0,0	0,2	1,8	3,2	0,0	0,1	0,7
101	STZGRSRGRSGRSGS	filaggrin	C	0,8	1,2	2,1	0,8	0,1	0,9	1,1	2,2	0,5	0,2	1,7	0,3	0,1	0,8	0,0	0,1	1,9	3,1	0,1	0,2	0,6
102	STZGZSRGRSGRSGS	filaggrin	C	1,0	1,0	1,6	1,3	0,1	0,8	1,2	2,1	0,6	0,3	1,6	0,4	0,1	0,6	0,2	0,0	2,0	3,8	0,0	0,1	0,1
103	STZGZSZGRSGRSGS	filaggrin	C	1,1	0,9	1,8	1,2	0,1	0,9	1,1	2,3	0,6	0,2	1,5	0,5	0,0	0,3	0,0	0,1	1,9	4,0	0,0	0,0	0,2
104	STZGZSZGZSGRSGS	filaggrin	C	1,2	0,8	1,7	1,2	0,1	0,9	1,1	1,0	1,1	0,9	1,5	0,5	0,0	1,4	1,1	0,2	1,8	4,2	0,0	0,0	0,1
105	STZGZSZGZSGZSGS	filaggrin	C	0,9	1,1	2,0	0,8	0,2	0,8	1,2	2,0	0,5	0,5	1,5	0,5	0,3	0,9	0,2	0,2	1,8	3,9	0,0	0,0	0,1
106	AIRRLARRGGVKRIS	histon 4	R	1,5	0,5	1,6	1,0	0,4	0,8	1,2	1,2	0,9	1,0	1,4	0,6	0,5	0,8	1,0	0,4	1,6	2,0	0,2	1,3	0,6
107	AIRRLARRGGVKZIS	histon 4	C	1,5	0,5	1,4	1,1	0,5	0,9	1,1	1,2	0,9	0,9	1,4	0,6	0,6	1,1	0,8	0,5	1,5	2,0	0,2	1,0	0,8
108	AIRRLARZGGVKRIS	histon 4	C	1,6	0,4	1,5	1,0	0,5	0,9	1,1	1,4	0,9	0,8	1,5	0,5	0,5	1,1	0,9	0,5	1,5	2,5	0,0	0,8	0,9
109	AIRRLAZRGGVKRIS	histon 4	C	1,5	0,5	1,6	0,9	0,4	1,0	1,0	1,4	0,9	0,7	1,4	0,6	0,6	1,1	0,6	0,5	1,5	3,0	0,0	0,4	0,6
110	AIRRLAZRGGVKZIS	histon 4	C	1,5	0,5	1,8	0,7	0,5	0,7	1,3	1,5	0,8	0,7	1,4	0,6	0,4	1,5	0,8	0,5	1,5	2,6	0,0	0,5	0,9
111	AIRRLAZZGGVKRIS	histon 4	C	1,3	0,7	1,7	0,8	0,4	1,0	1,0	1,8	0,5	0,7	1,5	0,5	0,4	1,3	0,9	0,4	1,6	2,6	0,0	0,5	0,9
112	AIRZLARRGGVKRIS	histon 4	C	1,3	0,7	1,9	0,8	0,3	1,0	1,0	1,5	0,8	0,8	1,5	0,5	0,6	0,9	1,1	0,4	1,6	2,6	0,0	0,8	0,6
113	AIRZLARRGGVKZIS	histon 4	C	1,4	0,6	1,6	0,9	0,5	0,7	1,3	1,4	0,8	0,9	1,4	0,6	0,6	1,0	1,1	0,4	1,6	1,9	0,2	1,0	1,0
114	AIRZLARZGGVKRIS	histon 4	C	1,7	0,3	1,6	0,9	0,4	1,0	1,0	1,5	0,7	0,8	1,4	0,6	0,5	1,0	1,1	0,4	1,6	2,3	0,0	0,8	0,9
115	AIRZLAZRGGVKRIS	histon 4	C	1,6	0,4	1,8	0,8	0,4	1,0	1,0	1,5	0,9	0,6	1,4	0,6	0,4	1,5	0,7	0,2	1,8	2,8	0,0	0,3	1,0
116	AIZRLARRGGVKRIS	histon 4	C	1,3	0,7	1,8	1,0	0,3	0,9	1,1	1,5	0,8	0,7	1,4	0,6	0,3	1,3	0,8	0,6	1,4	2,7	0,0	0,6	0,7
117	AIZRLARRGGVKZIS	histon 4	C	1,6	0,4	1,6	0,9	0,5	0,7	1,3	1,4	0,8	0,7	1,4	0,6	0,5	1,2	0,9	0,5	1,5	2,6	0,0	0,6	0,9
118	AIZRLARZGGVKRIS	histon 4	C	1,5	0,5	1,6	1,1	0,3	1,0	1,0	1,5	0,7	0,8	1,5	0,5	0,6	1,4	0,4	0,3	1,7	2,4	0,0	0,5	1,1
119	AIZRLAZRGGVKRIS	histon 4	C	1,4	0,6	1,7	0,9	0,4	0,9	1,1	1,5	0,8	0,8	1,5	0,5	0,7	1,2	0,7	0,3	1,7	2,4	0,0	0,4	1,2

120	AIZZLARRGGVKRIS	histon 4	C	1,4	0,6	1,6	1,1	0,4	0,9	1,1	1,4	0,7	0,9	1,4	0,6	0,4	1,1	0,9	0,5	1,5	1,9	0,0	1,1	1,0
121	AIZZLAZRGGVKRIS	histon 4	C	1,4	0,6	1,6	0,7	0,7	1,0	1,0	1,7	0,7	0,6	1,5	0,5	0,5	1,3	0,8	0,2	1,8	2,4	0,0	0,4	1,2
122	AIZZLAZZGGVKRIS	histon 4	C	1,3	0,7	1,6	1,0	0,5	0,8	1,2	2,0	0,4	0,6	1,4	0,6	1,0	0,4	1,0	0,4	1,6	1,6	0,0	1,1	1,3
123	AIZZLAZZGGVKZIS	histon 4	C	1,3	0,7	1,6	1,0	0,4	0,8	1,2	2,4	0,3	0,3	1,3	0,7	0,3	0,8	0,6	0,3	1,7	3,3	0,0	0,1	0,5
124	GAKRHRKVLRDNIQG	histon 4	R	0,9	1,1	1,9	0,9	0,2	0,8	1,2	1,7	0,7	0,6	1,4	0,6	1,1	0,4	1,0	0,4	1,6	0,7	0,5	1,4	1,3
125	GAKRHRKVLZDNIQG	histon 4	C	1,3	0,7	1,9	0,8	0,3	1,0	1,0	1,5	1,0	0,5	1,3	0,7	1,2	0,5	0,8	0,3	1,7	0,5	0,1	1,3	2,1
126	GAKRHZKVLRDNIQG	histon 4	C	1,2	0,8	2,1	0,5	0,4	1,0	1,0	1,4	1,1	0,5	1,4	0,6	1,1	0,7	1,0	0,2	1,8	0,5	0,3	1,0	2,2
127	GAKRHZKVLZDNIQG	histon 4	C	1,5	0,5	1,4	0,7	0,9	1,0	1,0	1,3	1,1	0,6	1,4	0,6	1,3	0,6	1,0	0,3	1,7	0,5	0,2	1,4	1,9
128	GAKZHRKVLRDNIQG	histon 4	C	1,3	0,7	2,1	0,6	0,3	1,1	0,9	1,4	1,0	0,5	1,4	0,6	0,9	0,6	1,1	0,3	1,7	0,5	0,2	1,1	2,3
129	GAKZHRKVLZDNIQG	histon 4	C	1,5	0,5	1,4	0,7	0,9	1,1	0,9	1,3	1,2	0,5	1,3	0,7	1,0	0,9	0,9	0,3	1,7	0,5	0,2	1,1	2,2
130	GAKZHZKVLRDNIQG	histon 4	C	0,9	1,1	2,1	0,5	0,5	0,9	1,1	1,3	1,2	0,5	1,5	0,5	1,2	0,7	1,0	0,3	1,7	0,3	0,2	1,7	1,8
131	GAKZHZKVLZDNIQG	histon 4	C	1,5	0,5	1,5	0,8	0,6	0,9	1,1	1,2	0,9	0,8	1,4	0,6	1,2	0,6	0,9	0,2	1,8	0,4	0,2	1,3	2,0
132	LRVTRGSRAPVSRAQ	proteoglycan	R	0,8	1,2	1,8	0,9	0,3	0,9	1,1	2,1	0,6	0,4	1,5	0,5	0,0	2,1	0,2	0,3	1,7	3,2	0,0	0,1	0,7
133	LRVTZGSRAPVSRAQ	proteoglycan	C	0,6	1,4	1,7	0,9	0,4	0,9	1,1	2,1	0,5	0,5	1,6	0,4	0,2	1,5	0,2	0,3	1,7	2,5	0,0	0,1	1,4
134	MDMCSAGWLADRSVR	proteoglycan	R	0,6	1,4	1,8	0,9	0,4	1,1	0,9	1,7	0,7	0,6	1,2	0,8	0,5	1,5	0,8	0,3	1,7	1,7	0,2	0,8	1,3
135	MDMCSAGWLADRSVZ	proteoglycan	C	0,8	1,2	1,9	0,9	0,2	1,0	1,0	2,2	0,4	0,4	1,4	0,6	0,4	0,9	0,4	0,1	1,9	3,1	0,0	0,1	0,8
136	MDMCSAGWLADZSVR	proteoglycan	C	0,9	1,1	2,0	1,0	0,1	0,9	1,1	2,6	0,2	0,2	1,1	0,9	0,4	0,5	0,3	0,0	2,0	2,1	0,0	0,7	1,1
137	MDMCSAGWLADZSVZ	proteoglycan	C	0,6	1,4	1,9	1,0	0,2	0,8	1,2	2,2	0,4	0,4	1,4	0,6	0,4	0,7	0,2	0,0	2,0	3,6	0,0	0,0	0,3
138	AYVTRSSAVRLRSSV	vimentin	R	1,3	0,7	2,3	0,6	0,1	1,8	0,2	1,7	0,7	0,7	1,4	0,6	0,5	1,0	0,5	0,7	1,3	2,9	0,0	0,3	1,0
139	AYVTRSSAVRLZSSV	vimentin	C	1,1	0,9	2,3	0,5	0,1	0,9	1,1	2,1	0,3	0,6	1,4	0,6	0,5	1,3	0,5	1,6	0,4	2,4	0,2	0,5	1,0
140	AYVTRSSAVZLRSSV	vimentin	C	0,9	1,1	2,4	0,5	0,1	0,8	1,2	2,0	0,3	0,7	1,5	0,5	0,5	1,1	0,9	0,4	1,6	2,4	0,1	0,3	1,2
141	AYVTRSSAVZLZSSV	vimentin	C	0,9	1,1	2,4	0,4	0,2	1,0	1,0	2,2	0,4	0,4	1,5	0,5	0,5	0,8	0,5	0,2	1,8	2,8	0,0	0,2	1,0
142	AYVTRSSAVZLZSSVP	vimentin	C	1,1	0,9	2,2	0,6	0,2	1,1	0,9	2,2	0,4	0,5	1,4	0,6	0,4	0,6	1,1	0,5	1,5	1,9	0,1	0,5	1,4
143	AYVTZSSAVRLRSSV	vimentin	C	1,1	0,9	2,3	0,6	0,2	0,7	1,3	1,8	0,6	0,6	1,5	0,5	0,6	0,7	1,4	0,8	1,2	2,1	0,2	0,8	0,9
144	AYVTZSSAVRLZSSV	vimentin	C	1,0	1,0	2,3	0,6	0,1	0,6	1,4	2,3	0,3	0,4	1,5	0,5	0,5	0,8	0,8	0,4	1,6	2,1	0,1	0,6	1,1
145	AYVTZSSAVZLRSSV	vimentin	C	1,1	0,9	2,3	0,6	0,1	0,6	1,4	2,3	0,2	0,5	1,4	0,6	0,7	0,5	1,1	0,4	1,6	2,5	0,0	0,4	1,1
146	AYVTZSSAVZLZSSV	vimentin	C	1,2	0,8	2,4	0,6	0,1	0,9	1,1	2,2	0,4	0,4	1,4	0,6	0,7	0,6	0,7	0,3	1,7	2,5	0,3	0,4	0,8
147	AYVTZSSAVZLZSSVP	vimentin	C	1,0	1,0	2,2	0,8	0,1	1,0	1,0	2,5	0,2	0,3	1,4	0,6	0,7	0,7	0,4	0,3	1,7	1,7	0,1	0,8	1,5
148	SAVRLRSSVPGVRLL	vimentin	R	1,1	0,9	1,8	0,9	0,3	1,0	1,0	1,6	0,6	0,8	1,4	0,6	0,4	1,3	0,6	1,4	0,6	0,2	3,5	0,1	0,1
149	SAVRLRSSVPGVZLL	vimentin	C	1,0	1,0	1,5	1,0	0,5	0,9	1,1	1,9	0,5	0,6	1,4	0,6	0,4	1,0	0,7	1,7	0,3	0,7	3,0	0,2	0,2
150	SAVRLZSSVPGVRLL	vimentin	C	1,2	0,8	1,6	0,9	0,5	0,8	1,2	1,6	0,7	0,7	1,4	0,6	0,4	1,0	0,9	1,5	0,5	0,7	2,8	0,2	0,3
151	SAVRLZSSVPGVZLL	vimentin	C	1,1	0,9	1,7	1,0	0,3	0,9	1,1	2,1	0,4	0,5	1,5	0,5	0,7	0,8	1,0	1,5	0,5	0,4	3,1	0,2	0,3
152	SAVZLRSSVPGVRLL	vimentin	C	1,0	1,0	1,6	0,8	0,6	1,0	1,0	1,4	0,7	0,8	1,4	0,6	0,5	1,2	0,8	1,6	0,4	1,7	0,9	0,5	0,9
153	SAVZLRSSVPGVZLL	vimentin	C	1,0	1,0	1,8	0,9	0,3	1,1	0,9	2,1	0,4	0,5	1,3	0,7	0,8	0,8	0,9	1,6	0,4	1,1	1,6	0,5	0,7
154	SAVZLZSSVPGVRLL	vimentin	C	1,2	0,8	1,6	1,0	0,4	0,9	1,1	1,9	0,5	0,6	1,4	0,6	0,6	0,7	1,1	1,7	0,3	0,5	2,6	0,3	0,6
155	SAVZLZSSVPGVZLL	vimentin	C	1,1	0,9	1,7	1,2	0,2	0,9	1,1	2,2	0,3	0,6	1,4	0,6	0,5	0,9	0,8	1,6	0,4	1,6	1,0	0,3	1,1
156	STRSVSSSSYRRMFG	vimentin	R	1,3	0,7	1,6	1,0	0,4	1,1	0,9	1,7	0,7	0,6	1,4	0,6	0,3	1,3	0,9	0,7	1,3	3,2	0,0	0,2	0,6
157	STRSVSSSSYRZMFG	vimentin	C	1,4	0,6	1,8	0,7	0,5	1,1	0,9	1,9	0,6	0,6	1,2	0,8	0,6	1,1	1,0	0,7	1,3	2,7	0,4	0,2	0,7
158	STRSVSSSSYZRMFG	vimentin	C	1,5	0,5	1,4	0,9	0,6	1,1	0,9	1,8	0,6	0,6	1,2	0,8	0,6	1,1	1,0	0,8	1,2	2,8	0,3	0,2	0,7
159	STRSVSSSSYZZMFG	vimentin	C	1,4	0,6	1,9	0,7	0,4	0,9	1,1	2,3	0,3	0,4	1,1	0,9	0,9	0,9	0,8	0,8	1,2	2,1	0,4	0,3	1,1
160	STZSVSSSSYRRMFG	vimentin	C	1,4	0,6	1,5	1,0	0,5	1,0	1,0	1,8	0,6	0,6	1,3	0,7	0,5	0,9	1,2	0,7	1,3	2,8	0,3	0,3	0,6
161	STZSVSSSSYRZMFG	vimentin	C	1,1	0,9	2,1	0,6	0,3	0,9	1,1	2,1	0,3	0,6	1,3	0,7	0,2	0,9	1,3	0,8	1,2	2,3	0,4	0,5	0,9
162	STZSVSSSSYZRMFG	vimentin	C	1,1	0,9	2,0	0,6	0,4	0,8	1,2	2,0	0,4	0,6	1,3	0,7	0,4	1,3	0,8	0,6	1,4	2,5	0,4	0,3	0,8
163	STZSVSSSSYZZMFG	vimentin	C	1,5	0,5	2,2	0,5	0,3	0,9	1,1	2,4	0,3	0,3	1,1	0,9	0,5	0,7	0,5	0,7	1,3	2,4	0,1	0,4	1,1
164	YVTRSSAVRLRSSVP	vimentin	R	1,1	0,9	2,2	0,6	0,2	1,0	1,0	1,4	0,7	0,9	1,5	0,5	0,4	1,2	0,7	1,2	0,8	2,2	0,0	0,8	1,3
165	YVTRSSAVRLZSSVP	vimentin	C	1,0	1,0	2,2	0,6	0,3	0,9	1,1	1,7	0,6	0,7	1,5	0,5	0,5	1,3	0,7	1,4	0,6	1,8	0,1	0,8	1,4
166	YVTRSSAVZLRSSVP	vimentin	C	0,8	1,2	2,2	0,6	0,2	0,9	1,1	1,8	0,5	0,6	1,5	0,5	0,7	1,1	0,7	0,0	4,7	1,5	0,2	1,0	1,4
167	YVTZSSAVRLRSSVP	vimentin	C	0,9	1,1	2,2	0,6	0,3	0,8	1,2	1,9	0,6	0,6	1,5	0,5	0,6	0,8	1,3	7,7	0,0	1,3	0,3	1,0	1,4
168	YVTZSSAVRLZSSVP	vimentin	C	0,9	1,1	2,1	0,6	0,2	0,8	1,2	2,2	0,4	0,4	1,4	0,6	0,7	0,8	1,0	1,0	1,0	1,5	0,1	0,9	1,5
169	YVTZSSAVZLRSSVP	vimentin	C	0,9	1,1	2,3	0,4	0,2	0,8	1,2	2,1	0,3	0,6	1,5	0,5	0,6	0,6	1,4	0,4	1,6	1,6	0,2	0,7	1,5

Positive sera to arginine peptide
Positive sera to citrullinated peptide
Positive sera specific to citrullinated peptide
