# Supplementary material for: PAD4 Immunization Triggers Anti-Citrullinated Peptide Antibodies in Normal Mice: Analysis With Peptide Arrays
Source: Front Immunol. 2022 Mar 31;13:840035. doi: 10.3389/fimmu.2022.840035 (PMC9008206; doi:10.3389/fimmu.2022.840035)
Supplement: Supplementary file 3 [file Image_3.pdf]

Supplementary figure 3: Immunization protocol and antibody analysis kinetic

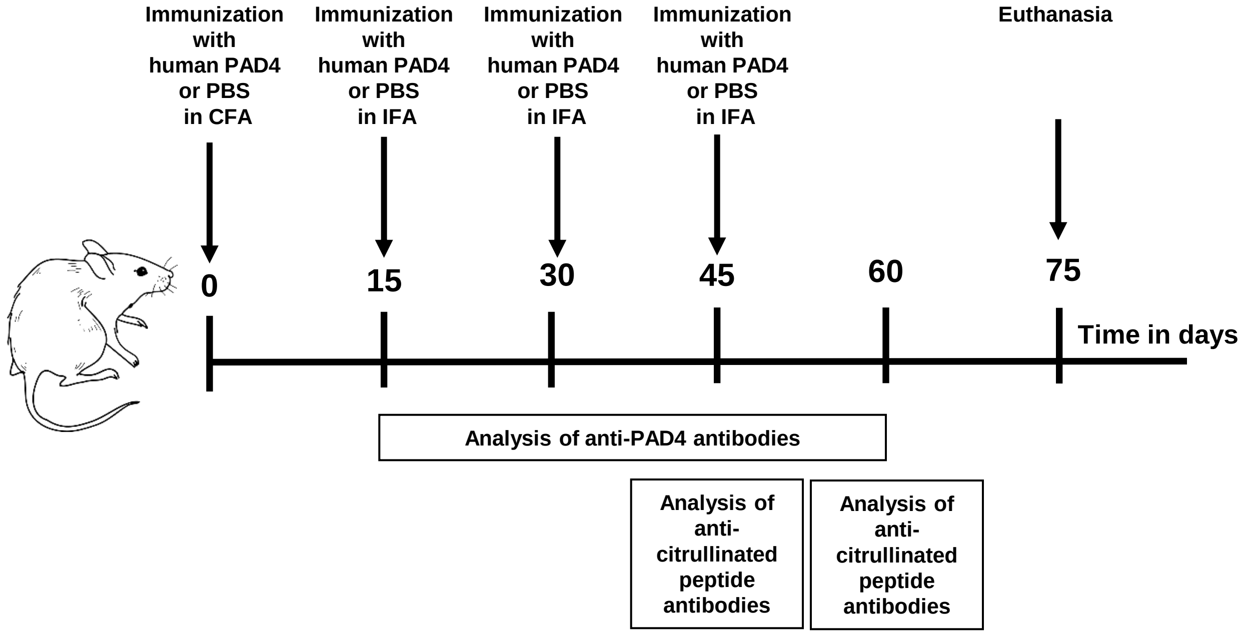

PAD4 : Peptidyl arginine deiminase
PBS: Phosphate buffered saline
CFA: Freund's complete adjuvant
IFA: Freund's incomplete adjuvant
