# Supplementary material for: PAD4 Immunization Triggers Anti-Citrullinated Peptide Antibodies in Normal Mice: Analysis With Peptide Arrays
Source: Front Immunol. 2022 Mar 31;13:840035. doi: 10.3389/fimmu.2022.840035 (PMC9008206; doi:10.3389/fimmu.2022.840035)
Supplement: Supplementary file 4 [file Image_4.pdf]

Supplementary figure 4 : Major peptide epitopes after hPAD4 immunization in different mouse strains. Peptides recognized by 60% of hPAD4 immunized mice and no PBS immunized mice were identified in BALB/c mice (A), BL6 mice (B) and C3H mice (C). A positive serum was defined by a ratio higher than 3.

A/

Peptide number	Peptide sequencce	Protein	Arginine (R) or citrulline (C)	BALB-1	BALB-2	BALB-3	Number of positive BALB/C mice
80	VVWMNWKGSWYSMRZ	beta fibrinogen	C	5,9	0,7	3,7	2

B/

Peptide number	Peptide sequencce	Protein	Arginine (R) or citrulline (C)	BL6-1	BL6-2	BL6-3	BL6-4	BL6-5	BL6-6	BL6-7	BL6-8	Number of positive BL6 mice
9	RIHAREIFDSRGNPT	enolase	R	15,3	3,4	3,4	0,5	1,0	3,3	3,2	0,5	5
19	GGGVRGPRVVERHQS	alpha fibrinogen	R	20,1	4,7	6,5	1,0	2,5	7,8	10,3	0,5	5
21	GGGVRGPZVVERHQS	alpha fibrinogen	C	12,9	4,9	18,9	1,2	1,2	4,4	5,9	0,5	5
23	GGGVZGPRVVERHQS	alpha fibrinogen	C	16,1	3,1	16,4	0,9	1,3	4,3	4,2	0,3	5
44	FSTYDZDNDGWVTTD	beta fibrinogen	C	2,8	3,1	3,6	2,4	3,5	1,9	5,8	11,7	5
87	WYNZCHAANPNGZYY	beta fibrinogen	C	3,6	0,6	0,5	8,1	7,8	3,7	7,7	0,5	5
95	WYSMZZMSMKIZPFF	beta fibrinogen	C	11,4	3,1	3,1	2,2	2,3	4,8	4,6	0,9	5
111	AIRRLAZZGGVKRIS	histon 4	C	25,6	3,8	4,8	1,1	2,0	11,3	7,3	0,6	5
115	AIRZLAZRGGVKRIS	histon 4	C	20,3	3,4	4,0	1,3	1,6	9,2	8,2	0,4	5
119	AIZRLAZRGGVKRIS	histon 4	C	16,6	3,1	3,5	1,1	1,6	8,3	8,5	0,5	5
121	AIZZLAZRGGVKRIS	histon 4	C	23,3	4,6	5,1	1,0	1,2	6,7	6,4	0,1	5
157	STRSVSSSSYRZMFG	vimentin	C	14,5	3,7	3,8	1,8	2,6	4,8	4,7	0,4	5
158	STRSVSSSSYZRMFG	vimentin	C	24,0	3,0	3,5	2,6	2,2	7,4	4,6	0,1	5

C/

Peptide number	Peptide sequencce	Protein	Arginine (R) or citrulline (C)	C3H-1	C3H-2	C3H-3	C3H-4	C3H-5	Number of positive C3H mice
2	GAZGLTGNPGVQGPE	collagen	C	1,3	3,1	3,1	5,7	3,0	3
35	SCSRAVNREINLQDY	alpha fibrinogen	R	0,5	3,5	1,1	3,5	3,1	3
55	MRZMSMKIZPFFPQQ	beta fibrinogen	C	1,6	3,1	2,1	5,1	3,1	3
57	MZRMSMKIZPFFPQQ	beta fibrinogen	C	2,7	4,2	3,4	6,0	3,3	4
58	MZZMSMKIRPFFPQQ	beta fibrinogen	C	2,9	4,1	3,3	3,7	2,5	3
106	AIRRLARRGGVKRIS	histon 4	R	3,3	3,0	9,8	3,2	2,3	3
107	AIRRLARRGGVKZIS	histon 4	C	5,3	3,8	10,5	3,7	2,6	4
108	AIRRLARZGGVKRIS	histon 4	C	5,1	4,0	10,4	4,0	2,6	4
110	AIRRLAZRGGVKZIS	histon 4	C	4,7	4,3	8,0	3,6	2,7	4
112	AIRZLARRGGVKRIS	histon 4	C	4,1	3,2	8,1	3,1	2,5	4
113	AIRZLARRGGVKZIS	histon 4	C	6,7	4,5	12,1	3,5	2,5	4
114	AIRZLARZGGVKRIS	histon 4	C	8,9	5,6	14,7	3,4	2,5	4
115	AIRZLAZRGGVKRIS	histon 4	C	5,5	4,7	10,4	3,4	2,6	4
116	AIZRLARRGGVKRIS	histon 4	C	3,7	3,3	8,2	3,1	2,4	4
117	AIZRLARRGGVKZIS	histon 4	C	3,7	4,0	8,8	4,2	3,1	5
118	AIZRLARZGGVKRIS	histon 4	C	3,7	3,3	6,8	3,1	2,5	4
119	AIZRLAZRGGVKRIS	histon 4	C	3,9	3,4	6,9	3,2	2,6	4
120	AIZZLARRGGVKRIS	histon 4	C	6,5	4,5	11,6	2,7	2,2	3
121	AIZZLAZRGGVKRIS	histon 4	C	4,2	4,7	8,3	2,9	2,0	3
122	AIZZLAZZGGVKRIS	histon 4	C	2,0	5,2	7,8	3,3	2,1	3
138	AYVTRSSAVRLRSSV	vimentin	R	5,1	3,8	6,9	2,7	3,5	4
139	AYVTRSSAVRLZSSV	vimentin	C	3,1	4,8	4,9	2,2	3,2	4
140	AYVTRSSAVZLRSSV	vimentin	C	2,1	3,6	3,8	1,9	3,6	3
143	AYVTZSSAVRLRSSV	vimentin	C	3,5	4,8	4,8	2,0	2,9	3
146	AYVTZSSAVZLZSSV	vimentin	C	1,3	6,9	2,7	3,0	4,5	3
149	SAVRLRSSVPGVZLL	vimentin	C	2,8	3,4	4,1	3,2	2,3	3
154	SAVZLZSSVPGVRLL	vimentin	C	1,4	3,6	3,3	3,9	2,5	3
157	STRSVSSSSYRZMFG	vimentin	C	4,1	4,3	4,7	1,7	3,5	4
158	STRSVSSSSYZRMFG	vimentin	C	4,6	4,3	6,1	2,2	3,9	4
159	STRSVSSSSYZZMFG	vimentin	C	1,9	3,5	3,1	2,2	4,7	3
160	STZSVSSSSYRRMFG	vimentin	C	3,9	3,8	4,8	2,3	4,4	4
164	YVTRSSAVRLRSSVP	vimentin	R	3,4	2,9	4,5	2,4	3,7	3

Positive sera to arginine peptide
Positive sera to citrullined peptide
Positive sera specific to citrullinated peptide
