# Supplementary material for: PAD4 Immunization Triggers Anti-Citrullinated Peptide Antibodies in Normal Mice: Analysis With Peptide Arrays
Source: Front Immunol. 2022 Mar 31;13:840035. doi: 10.3389/fimmu.2022.840035 (PMC9008206; doi:10.3389/fimmu.2022.840035)
Supplement: Supplementary file 5 [file Table_1.pdf]

Supplementary table 1: Peptides in peptide arrays

Peptide number	Sequence	Protein	Arginine (R) citrulline (C)	Origin
1	GARGLTGNPGVQGPE	collagen	R	H
2	GAZGLTGNPGVQGPE	collagen	C	H
3	SRGQSRGRGRGRGRG	ebna 2	R	NA
4	SZGQSZGZGZGZGZG	ebna 2	C	NA
5	IHAREIFDSRGNPTV	enolase	R	H/M
6	IHAREIFDSZGNPTV	enolase	C	H/M
7	IHAZEIFDSRGNPTV	enolase	C	H/M
8	IHAZEIFDSZGNPTV	enolase	C	H/M
9	RIHAREIFDSRGNPT	enolase	R	M
10	RIHAREIFDSZGNPT	enolase	C	M
11	RIHAZEIFDSRGNPT	enolase	C	M
12	RIHAZEIFDSZGNPT	enolase	C	M
13	TAKGLFRAAVPSGAS	enolase	R	H
14	TAKGLFZAAVPSGAS	enolase	C	H
15	ZIHAREIFDSRGNPT	enolase	C	M
16	ZIHAREIFDSZGNPT	enolase	C	M
17	ZIHAZEIFDSRGNPT	enolase	C	M
18	ZIHAZEIFDSZGNPT	enolase	C	M
19	GGGVRGPRVVERHQS	alpha fibrinogen	R	H/M
20	GGGVRGPRVVEZHQS	alpha fibrinogen	C	H/M
21	GGGVRGPZVVERHQS	alpha fibrinogen	C	H/M
22	GGGVRGPZVVEZHQS	alpha fibrinogen	C	H/M
23	GGGVZGPRVVERHQS	alpha fibrinogen	C	H/M
24	GGGVZGPRVVEZHQS	alpha fibrinogen	C	H/M
25	GGGVZGPZVVERHQS	alpha fibrinogen	C	H/M
26	GGGVZGPZVVEZHQS	alpha fibrinogen	C	H/M
27	GPRVVERHQSQCKDS	alpha fibrinogen	R	M
28	GPRVVEZHQSQCKDS	alpha fibrinogen	C	M
29	GPZVVERHQSQCKDS	alpha fibrinogen	C	M
30	GPZVVEZHQSQCKDS	alpha fibrinogen	C	M
31	MELERPGKDGGSRGD	alpha fibrinogen	R	M
32	MELERPGKDGGSZGD	alpha fibrinogen	C	M
33	MELEZPGKDGGSRGD	alpha fibrinogen	C	M
34	MELEZPGKDGGSZGD	alpha fibrinogen	C	M
35	SCSRAVNREINLQDY	alpha fibrinogen	R	M
36	SCSRAVNZEINLQDY	alpha fibrinogen	C	M
37	SCSZAVNREINLQDY	alpha fibrinogen	C	M
38	SCSZAVNZEINLQDY	alpha fibrinogen	C	M
39	APPPISGGGYRARPA	beta fibrinogen	R	H/M
40	APPPISGGGYRAZPA	beta fibrinogen	C	H/M
41	APPPISGGGYZARPA	beta fibrinogen	C	H/M
42	APPPISGGGYZAZPA	beta fibrinogen	C	H/M
43	FSTYDRDNDGWVTTD	beta fibrinogen	R	M
44	FSTYDZDNDGWVTTD	beta fibrinogen	C	M
45	GSWYSMRRMSMKIRP	beta fibrinogen	R	M
46	GSWYSMRRMSMKIZP	beta fibrinogen	C	M
47	GSWYSMRZMSMKIRP	beta fibrinogen	C	M
48	GSWYSMZRMSMKIRP	beta fibrinogen	C	M
49	GSWYSMZZMSMKIZP	beta fibrinogen	C	M
50	LVGENRTMTIHNGMF	beta fibrinogen	R	M
51	LVGENZTMTIHNGMF	beta fibrinogen	C	M
52	MRRMSMKIRPFFPQQ	beta fibrinogen	R	M
53	MRRMSMKIZPFFPQQ	beta fibrinogen	C	M
54	MRZMSMKIRPFFPQQ	beta fibrinogen	C	M
55	MRZMSMKIZPFFPQQ	beta fibrinogen	C	M
56	MZRMSMKIRPFFPQQ	beta fibrinogen	C	M
57	MZRMSMKIZPFFPQQ	beta fibrinogen	C	M
58	MZZMSMKIRPFFPQQ	beta fibrinogen	C	M
59	MZZMSMKIZPFFPQQ	beta fibrinogen	C	M
60	NRCHAANPNGRYYWG	beta fibrinogen	R	H/M
61	NRCHAANPNGZYYWG	beta fibrinogen	C	H/M
62	NZCHAANPNGRYYWG	beta fibrinogen	C	H/M
63	NZCHAANPNGZYYWG	beta fibrinogen	C	H/M
64	PRKQCSKEDGGGWWY	beta fibrinogen	R	H/M
65	PZKQCSKEDGGGWWY	beta fibrinogen	C	H/M
66	QKLESDISAQMEYCR	beta fibrinogen	R	M
67	QKLESDISAQMEYCZ	beta fibrinogen	C	M
68	RPAPPPISGGGYRAR	beta fibrinogen	R	H/M
69	RPAPPPISGGGYRAZ	beta fibrinogen	C	H/M
70	RPAPPPISGGGYZAR	beta fibrinogen	C	H/M
71	RPAPPPISGGGYZAZ	beta fibrinogen	C	H/M
72	VIQNRQDGSVDFGRK	beta fibrinogen	R	H/M
73	VIQNRQDGSVDFGZK	beta fibrinogen	C	H/M
74	VIQNZQDGSVDFGRK	beta fibrinogen	C	H/M
75	VIQNZQDGSVDFGZK	beta fibrinogen	C	H/M
76	VTTDPRKQCSKEDGG	beta fibrinogen	R	M
77	VTTDPZKQCSKEDGG	beta fibrinogen	C	M
78	VVWMNWKGSWYSMRK	beta fibrinogen	R	H
79	VVWMNWKGSWYSMRR	beta fibrinogen	R	M
80	VVWMNWKGSWYSMRZ	beta fibrinogen	C	M
81	VVWMNWKGSWYSMZK	beta fibrinogen	C	H
82	VVWMNWKGSWYSMZR	beta fibrinogen	C	M
83	VVWMNWKGSWYSMZZ	beta fibrinogen	C	M
84	WYNRCHAANPNGRYY	beta fibrinogen	R	H/M
85	WYNRCHAANPNGZYY	beta fibrinogen	C	H/M
86	WYNZCHAANPNGRYY	beta fibrinogen	C	H/M

87	WYNZCHAANPNGZYY	beta fibrinogen	C	H/M
88	WYSMRRMSMKIRPFF	beta fibrinogen	R	M
89	WYSMRRMSMKIZPFF	beta fibrinogen	C	M
90	WYSMRZMSMKIRPFF	beta fibrinogen	C	M
91	WYSMRZMSMKIZPFF	beta fibrinogen	C	M
92	WYSMZRMSMKIRPFF	beta fibrinogen	C	M
93	WYSMZRMSMKIZPFF	beta fibrinogen	C	M
94	WYSMZZMSMKIRPFF	beta fibrinogen	C	M
95	WYSMZZMSMKIZPFF	beta fibrinogen	C	M
96	ZPAPPPISGGGYRAR	beta fibrinogen	C	H/M
97	ZPAPPPISGGGYRAZ	beta fibrinogen	C	H/M
98	ZPAPPPISGGGYZAR	beta fibrinogen	C	H/M
99	ZPAPPPISGGGYZAZ	beta fibrinogen	C	H/M
100	STRGRSRGRSGRSGS	filaggrin	R	H
101	STZGRSRGRSGRSGS	filaggrin	C	H
102	STZGZSRGRSGRSGS	filaggrin	C	H
103	STZGZSZGRSGRSGS	filaggrin	C	H
104	STZGZSZGZSGRSGS	filaggrin	C	H
105	STZGZSZGZSGZSGS	filaggrin	C	H
106	AIRRLARRGGVKRIS	histon 4	R	H/M
107	AIRRLARRGGVKZIS	histon 4	C	H/M
108	AIRRLARZGGVKRIS	histon 4	C	H/M
109	AIRRLAZRGGVKRIS	histon 4	C	H/M
110	AIRRLAZRGGVKZIS	histon 4	C	H/M
111	AIRRLAZZGGVKRIS	histon 4	C	H/M
112	AIRZLARRGGVKRIS	histon 4	C	H/M
113	AIRZLARRGGVKZIS	histon 4	C	H/M
114	AIRZLARZGGVKRIS	histon 4	C	H/M
115	AIRZLAZRGGVKRIS	histon 4	C	H/M
116	AIZRLARRGGVKRIS	histon 4	C	H/M
117	AIZRLARRGGVKZIS	histon 4	C	H/M
118	AIZRLARZGGVKRIS	histon 4	C	H/M
119	AIZRLAZRGGVKRIS	histon 4	C	H/M
120	AIZZLARRGGVKRIS	histon 4	C	H/M
121	AIZZLAZRGGVKRIS	histon 4	C	H/M
122	AIZZLAZZGGVKRIS	histon 4	C	H/M
123	AIZZLAZZGGVKZIS	histon 4	C	H/M
124	GAKRHRKVLRDNIQG	histon 4	R	H/M
125	GAKRHRKVLZDNIQG	histon 4	C	H/M
126	GAKRHZKVLRDNIQG	histon 4	C	H/M
127	GAKRHZKVLZDNIQG	histon 4	C	H/M
128	GAKZHRKVLRDNIQG	histon 4	C	H/M
129	GAKZHRKVLZDNIQG	histon 4	C	H/M
130	GAKZHZKVLRDNIQG	histon 4	C	H/M
131	GAKZHZKVLZDNIQG	histon 4	C	H/M
132	LRVTRGSRAPVSRAQ	proteoglycan	R	H/M
133	LRVTZGSRAPVSRAQ	proteoglycan	C	H/M
134	MDMCSAGWLADRSVR	proteoglycan	R	H/M
135	MDMCSAGWLADRSVZ	proteoglycan	C	H/M
136	MDMCSAGWLADZSVR	proteoglycan	C	H/M
137	MDMCSAGWLADZSVZ	proteoglycan	C	H/M
138	AYVTRSSAVRLRSSV	vimentin	R	M
139	AYVTRSSAVRLZSSV	vimentin	C	M
140	AYVTRSSAVZLRSSV	vimentin	C	M
141	AYVTRSSAVZLZSSV	vimentin	C	M
142	AYVTRSSAVZLZSSVP	vimentin	C	M
143	AYVTZSSAVRLRSSV	vimentin	C	M
144	AYVTZSSAVRLZSSV	vimentin	C	M
145	AYVTZSSAVZLRSSV	vimentin	C	M
146	AYVTZSSAVZLZSSV	vimentin	C	M
147	AYVTZSSAVZLZSSVP	vimentin	C	M
148	SAVRLRSSVPGVRLL	vimentin	R	H/M
149	SAVRLRSSVPGVZLL	vimentin	C	H/M
150	SAVRLZSSVPGVRLL	vimentin	C	H/M
151	SAVRLZSSVPGVZLL	vimentin	C	H/M
152	SAVZLRSSVPGVRLL	vimentin	C	H/M
153	SAVZLRSSVPGVZLL	vimentin	C	H/M
154	SAVZLZSSVPGVRLL	vimentin	C	H/M
155	SAVZLZSSVPGVZLL	vimentin	C	H/M
156	STRSVSSSSYRRMFG	vimentin	R	H/M
157	STRSVSSSSYRZMFG	vimentin	C	H/M
158	STRSVSSSSYZRMFG	vimentin	C	H/M
159	STRSVSSSSYZZMFG	vimentin	C	H/M
160	STZSVSSSSYRRMFG	vimentin	C	H/M
161	STZSVSSSSYRZMFG	vimentin	C	H/M
162	STZSVSSSSYZRMFG	vimentin	C	H/M
163	STZSVSSSSYZZMFG	vimentin	C	H/M
164	YVTRSSAVRLRSSVP	vimentin	R	M
165	YVTRSSAVRLZSSVP	vimentin	C	M
166	YVTRSSAVZLRSSVP	vimentin	C	M
167	YVTZSSAVRLRSSVP	vimentin	C	M
168	YVTZSSAVRLZSSVP	vimentin	C	M
169	YVTZSSAVZLRSSVP	vimentin	C	M

Z: citrulline
H: human sequence
M: murine sequence
H/M: identical human and murine sequences
